# A software package for efficient patient trajectory analysis applied to analyzing bladder cancer development

**DOI:** 10.1371/journal.pdig.0000384

**Published:** 2023-11-22

**Authors:** Charlotte Herzeel, Ellie D’Hondt, Valerie Vandeweerd, Wouter Botermans, Murat Akand, Frank Van der Aa, Roel Wuyts, Wilfried Verachtert

**Affiliations:** 1 ExaScience Lab, imec, Leuven, Belgium; 2 Janssen Research & Development, a division of Janssen Pharmaceutica NV, Beerse, Belgium; 3 Department of Urology, University Hospitals Leuven, Leuven, Belgium; Ben-Gurion University of the Negev, ISRAEL

## Abstract

We present the Patient Trajectory Analysis Library (PTRA), a software package for explorative analysis of patient development. PTRA provides the tools for extracting statistically relevant trajectories from the medical event histories of a patient population. These trajectories can additionally be clustered for visual inspection and identifying key events in patient progression. The algorithms of PTRA are based on a statistical method developed previously by Jensen et al, but we contribute several modifications and extensions to enable the implementation of a practical tool. This includes a new clustering strategy, filter mechanisms for controlling analysis to specific cohorts and for controlling trajectory output, a parallel implementation that executes on a single server rather than a high-performance computing (HPC) cluster, etc. PTRA is furthermore open source and the code is organized as a framework so researchers can reuse it to analyze new data sets. We illustrate our tool by discussing trajectories extracted from the TriNetX Dataworks database for analyzing bladder cancer development. We show this experiment uncovers medically sound trajectories for bladder cancer.

## Author summary

In this paper, we present a software tool for analyzing patient development over time. Our assumption is that, when looking at a patient population, there will be specific disease trajectories that frequently occur. Knowing general development trajectories is relevant for predicting patient progression over time, for example to decide on specific patient care. Our tool automatically extracts these trajectories from patient diagnosis and treatment histories available in their medical records. For such methods to work well we need to apply these analysis techniques on large databases. One of our technical contributions in this regard is developing new algorithms that can scale both in terms of computational performance as well as medical interpretability. We developed our tool in the context of a concrete use case to discover disease development trajectories for bladder cancer. The results are medically sound, which shows the value of the tool for discovering (unknown) patient development patterns. Additionally, our tool is released as an open source project, making it accessible for the entire research community.

## Introduction

Predicting patient progression is of key importance in medicine. Certain patterns, such as the co-occurrence of specific diseases, are well known. Patient progression and how co-morbid diseases influence this progression, are ongoing research. The expectation is that analyzing the medical histories of large populations can help to discover new insights in terms of patient progression over time [1].

In this context, a number of statistical and machine learning methods are being developed for discovering temporal disease trajectories in large-scale databases with electronically registered patient histories [1–9]. Specifically, these approaches investigate patient histories that consist of medical diagnoses in the form of International Classification of Diseases 9 or 10 (ICD-9 or ICD-10) codes [10] or other standardized medical description codes (SNOMED, READ, MedDRA) recorded for each doctor visit. The term “disease trajectory” then refers to a series of diagnoses in such a standardized format. The databases targeted by these analyses typically contain thousands of patients and span several years [1–9].

The trajectories found by these approaches confirm known information about patient progression [1, 3], but also enable generating and confirming new medical hypotheses. Examples of this include insights in terms of the effects of mis- and over-diagnosis on patient progression [8], the impact of certain treatments and drugs [8], the influence of diabetes on developing co-morbid diseases and the differences between the sexes in terms of the speed of development [5], the increased risk of cardiovascular disease progression for specific populations [4], and various others [1–6, 8].

It is currently difficult to reuse the methods proposed in the literature since they are closed source projects. Their mathematical descriptions are available in papers, but it is not trivial to reimplement them from such a description. For example, some methods claim requiring cluster computing [1], which requires advanced coding skills. Other methods use machine learning algorithms, but without source code and thus documentation on how to set parameters for these algorithms, reuse of the methods is challenging [3–5]. Our understanding is that the literature focuses on presenting medical discoveries made with temporal disease trajectories, but we argue there is value in being able to adapt the methods for analyzing new data sets or to reproduce their results. A notable exception is the work by Paik et al [7, 9], for which source code was released, but while they released an interesting GUI and visualization method, they only released a simplified method to calculate trajectories compared to the proposals in related work [1–5, 8] and do not provide any code for clustering trajectories.

In this paper we present PTRA, an open-source software package for analyzing temporal disease trajectories. The algorithms underlying PTRA are based on the method proposed by Jensen et al [1]. Reformulation of other methods within the PTRA framework is possible. We contribute several modifications and extensions to the Jensen method. We generalize the algorithms to work on any type of timestamped medical event rather than only ICD codes. This for example allows the tool to automatically remap ICD codes onto clinical categories (CCSR [11]) or to add treatments as medical events of interest in addition to diagnoses. We also propose a new clustering strategy which is capable of clustering all trajectories found for a population rather than only a subset (as in the Jensen method) which requires manual intervention by the user. We also added a filter mechanism to allow users to restrict trajectory building for specific patient groups or to control which kind of trajectories are reported, for example to focus on specific diseases. Finally, we take computational performance into account and reformulate algorithms as parallel algorithms, resulting in an efficient implementation that can run on a single multicore server.

We claim that the new features PTRA introduces, in combination with its accessibility as an open-source project, make it a valuable contribution to the research community. To validate our claim, we describe the use of PTRA to extract temporal disease trajectories from the TriNetX Dataworks database [12]. Concretely, we focus on analyzing disease progression in bladder cancer patients. We show that PTRA is able to extract trajectories for this population that match generally known patterns for an aging population such as bladder cancer patients. We also discuss the discovery of bladder cancer-specific trajectories. To the best of our knowledge, it is the first such computational analysis for bladder cancer data. We additionally report the results of the benchmarks that illustrate the computational efficiency of PTRA. Finally, we discuss the general organization of the PTRA package and how it can be reused to analyze new data sets, and implement new analysis techniques.

## Materials and methods

We next describe the PTRA software, the experiments with TriNetX, data sets, software versions, and hardware setups we use to evaluate the computational performance of PTRA.

### Implementation

PTRA is developed for Linux at the ExaScience Lab at imec. The PTRA software is written in Go, a programming language developed by Google, which we find to strike a good balance between ease of use and performance for implementing life science applications [13, 14]. The software is released as an open-source project on Github under the terms of the GNU Affero General Public License version 3 as published by the Free Software Foundation, with Additional Terms. See http://github.com/ExaScience/ptra.

### PTRA overview

The Patient TRajectory Analysis Library (PTRA) is a software package for analyzing medical event histories. It implements an algorithm that extracts statistically relevant trajectories from the medical event histories of a patient population.

For example, consider we have the diagnosis histories of a patient population shown in Listing 1:

1: *Cough*, *Broken leg*, *Dyspnea*, *Depression*, *Viral infection*, *Cough*, *COPD*, …

2: *Cough*, *BMI* > 30, *Wound*, *Cough*, *High blood pressure*, *Dyspnea*, *Diabetes*, *COPD*, …

3: *BMI* > 30, *Tooth ache*, *Pain*, *Wound*, *High blood pressure*, *Broken leg*, *Diabetes*, …

…

N: …

**Listing 1**. Mockup diagnosis histories.

We can see there exists overlap between histories in the population and can imagine that we can extract statically relevant trajectories, i.e. trajectories that frequently occur (Listing 2).

1: *Cough* → *Dyspnea* → *COPD*

2: *BMI* > 30 → *High blood pressure* → *Diabetes*

**Listing 2**. Mockup diagnosis trajectories.

A trajectory is defined as an ordered list of medical events that follow each other in time. For our mockup example, we have a trajectory where a “Cough” diagnosis precedes a “Dyspnea” diagnosis, which in turn is followed by a “COPD” diagnosis. Such trajectories are useful information for predicting patient progression over time [1]. Note that these type of temporal trajectories are different from time series. We want to model the subsequent occurrence of multiple different events. Instead, with time series, the progression of a single variable is modeled over time, such as for example the value of blood sugar levels throughout a day.

#### Algorithms

The algorithm for computing trajectories is based on the method for analyzing trajectories proposed by Jensen et al [1]. From a high level perspective, it consists of three steps:

1. Select medical event pairs *A* → *B* with a relative risk score (RR) > 1.0:
  - ∀ possible disease pairs *A* → *B* calculate the RR, i.e. the chance for developing B when diagnosed with A.
  - The RR is calculated via a sampling experiment comparing p-values to guarantee a fair estimation of the RR based on our data.
  - If both *A* → *B* > 1.0 and *B* → *A* > 1.0, perform a directionality test with a binomial experiment to select one pair.
  - Only keep the pairs *A* → *B* for which there is a minimum number of patients.
2. Build trajectories *A* → *B* → … → *Z* by chaining pairs:
  - E.g. *A* → *B* and *B* → *C* form a new trajectory *A* → *B* → *C*.
  - For each extension *A* → *B* → *C* check that there is a minimum number of patients.
3. Cluster trajectories for visual inspection, pattern detection, and hypothesis generation.

The details of these steps are discussed next.

#### 1. Selection of medical event pairs

The selection of medical event pairs requires calculating the relative risk scores for all possible pairs, i.e. calculating for each event pair *A* → *B* what the probability is that a medical event *B* follows a medical event *A* [1, 15]. For example, if the RR is 6, it means that B occurs 6x more for patients who have previously been diagnosed with A than those who have not been diagnosed with A.

The usual way to calculate relative risk scores is to set up a prospective cohort study comparing patients exposed to *A* with patients not exposed to *A* and then counting the occurrence of event *B* in both patient groups to calculate the probability for *A* → *B*. However, the idea here is to calculate the relative risk scores with the data available in the patient histories. The strategy is to setup random sampling experiments on this data to have a fair simulation of cohort studies.

Simplified pseudo code for calculating relative risk scores for all possible medical event pairs is shown in Listing 3.

∀ pair A→B:

 (P1 ← get patients exposed to A

 P2 ← sample *#P1 similar patients ¬exposed to A*

 Count #(*A*→*B*) *in P1, Count* #(¬*A*→*B*) *in P2*

 Calculate RR)

 × 10.000

Calculate p-value

**Listing 3**. Pseudo code relative risk score (RR) calculation.

For all possible event pairs (e.g. all combinations of ICD diagnosis codes), we calculate the number of patients exposed to a first medical event (*A*). Subsequently, we sample similar patients in terms of age, sex, localization, etc that are not exposed to that medical event (¬*A*). It is important to sample similar patients to avoid Simpson’s paradox [1]. For both patient groups, we count if a second event (*B*) follows the first medical event or absence thereof. Using these counts, we can calculate the relative risk score (RR). We repeat this experiment a number of times to calculate p-values and guarantee a fair random selection of patient groups to simulate the cohort study.

The repeated random sampling process to calculate RR scores is computationally expensive. The sampling loop needs to be processed for each possible combination of medical events. If we assume for example that the medical events are diagnoses encoded in ICD10, this means attempting a combination of 1064 × 1064 diagnosis codes for level 3 in de ICD10 hierarchy. The computation also relies on a random number generator to sample the patient groups (P2), which is computationally a costly operation.

We therefore parallelize the calculation of RR scores in the PTRA implementation. The RR computation for each pair *A* → *B* can occur independently. We can therefore split up the iteration space of our loop that calculates RRs and pass these iteration groups to independent parallel tasks. Profiling reveals that random number generation is computationally the most costly step in the RR calculation. We therefore use a custom library for random number generation rather than using the one from the Go standard library [16].

We refer to the source code [17] for more details on the RR calculation we left out in our discussion so far. For example, there is a statistical step for filtering *A* → *B* pairs upfront so they are not checked in the sampling experiment. For input data that is in ICD format, there are filters to remove entire ICD categories from the analysis (e.g. related to pregnancy). Also, counting *A* → *B* pairs must take into account timing constraints between diagnoses (e.g. maximum 5 years, minimum 6 months). For ICD codes, the ICD hierarchy must be taken into account as well as the level the analysis is run with.

#### 2. Building trajectories

The first step for building trajectories involves selecting medical event pairs based on the value of the RR score for that pair. The selected pairs are the starting points for chaining combinations of pairs into trajectories. By default, all pairs with a RR score >1.0 are selected for trajectory building. Additionally, if a pair *A* → *B* and its reverse *B* → *A* both have a RR score >1.0, a binomial experiment is used to test the directionality and select either the pair or its reverse for trajectory building. Finally, for a pair *A* → *B* to be considered for trajectory building, there also has to be a minimum number of patients in the population that are diagnosed with *A* → *B* [1].

To parallelize the trajectory building, the selected diagnosis pairs are divided among a number of parallel processes. These pairs are seen as partial trajectories. Each process subsequently attempts to extend its partial trajectories by trying to combine them with all of the selected pairs. For example, a partial trajectory *A* → *B* can be combined with *B* → *C* because they overlap with diagnosis *B*, provided additional constraints are satisfied. These constraints are 1) that the extended trajectory contains a minimum number of patients that follow it and 2) that the time between *A* → *B* and *B* → *C* satisfies a certain time span (e.g. between 6 months and 5 years). The extended trajectories found this way are subsequently checked for extension until no more extensions are possible. If extension is not possible, a partial trajectory is finalized and returned as a result.

Each process uses a thread-local stack for storing its intermediate trajectories. The finalized trajectories are also written to a thread-local buffer. After all processes are finished, local results are merged. The parallelism we describe here can be implemented with a fork/join pattern. Care must be taken that partial trajectories are copied when extended rather than destructively written since they may be extended multiple times. We found our fork/join-based parallelization to run efficiently on a single shared memory multicore server, whereas the Jensen method needs cluster computing for processing similarly sized data sets [1].

#### 3. Clustering trajectories

The Jensen algorithm for computing trajectories produces trajectories with a lot of overlap [1]. Therefore clustering of the trajectories is used to group together similar trajectories for visual inspection. The clusters are visualized by representing all trajectories in the clusters as a graph: diagnoses are the nodes of the graph and trajectory transitions are the edges. This type of visualization of trajectory clusters can help interpreting the trajectories and generating medical hypotheses [1–5].

The original algorithm by Jensen et al. clusters trajectories as shown in the pseudo code in Listing 4.

*#Cluster the event codes*

G ← make a graph: event codes (D) = nodes, RR pairs = edges

*#Place a similarity score on each edge ∈ G using the Jaccard index*:

∀ *Edge A* → *B* ∈ *G*:

 Jaccard(*A* → *B*)←(#*T*|*A* → *B* ∈ *T*)/(#*T*|*A* ∈ *T* + #*T*|*B* ∈ *T* − #*T*|*A* → *B* ∈ *T*)

*#Cluster G with MCL, assigns each T to a cluster C*_*i*_

*C*_1_, *C*_2_, …*C*_*N*_ ← *MCL*(*G*)

*# Assign each trajectory T to a cluster C*_*i*_

*T* ∈ *C*_*i*_ *if* ∀*D* ∈ *T*|*D* ∈ *C*_*i*_

**Listing 4**. Pseudo code clustering strategy in Jensen et al [1].

The strategy is to first cluster the event codes. A graph is created that places each event code onto a unique node. The selected pairs (from step 2) are used to create edges between event nodes. For each edge in the graph, a similarity score is calculated using the Jaccard index. As the pseudo code shows, the Jaccard index is calculated for a pair *A* → *B* by checking how many trajectories T exist that contain that pair and compare it to how many trajectories exist that contain its constituents *A* and *B*, but not the pair itself [1].

Once each edge is assigned a Jaccard index, the medical event graph is clustered using the MCL [18] algorithm. Finally, trajectories are assigned to their clusters. A trajectory is assigned to a cluster if all of the event codes used in the trajectory are a part of the cluster. In practice, this means certain trajectories are not assigned to any cluster, as event codes are unique in the graph [1]. The solution proposed by Jensen et al [1] is to manually merge clusters and assign trajectories that are otherwise not assigned to the merged clusters. We found that this method becomes impractical for analyzing specific data sets. For example, as we discuss in later sections, the strategy leaves up to 2/3 of the trajectories in TriNetX Dataworks to be assigned manually.

We propose a different clustering strategy than the one described by Jensen et al. Pseudo code is shown below in Listing 5.

*# Assign each trajectory T a unique ID*

G ← make a graph: trajectory IDs = nodes, fully connected

*# Place a similarity score on each edge ∈ G*

*# using the Jaccard index to directly compare T*

*# View T as sets of medical event codes*

∀ *Edge T*_1_ → *T*_2_ ∈ *G*:

 *Jaccard*(*T*_1_, *T*_2_ ← |*T*_1_ ∩ *T*_2_|/(|*T*_1_| + |*T*_2_| − |*T*_1_ ∩ *T*_2_|)

*# Cluster G with MCL, assigns each T to a cluster C*_*i*_

*C*_1_, *C*_2_, …*C*_*N*_ ← *MCL*(*G*)

*# Assign T to cluster C if TID ∈ C*

*T* ∈ *C*_*i*_ *if* *TID*(*T*)∈*C*_*i*_

**Listing 5**. Pseudo code PTRA clustering strategy.

The idea behind our clustering strategy is to directly cluster the trajectories. To do this, we assign each trajectory (T) a unique trajectory ID (TID). We make a graph that has as nodes all of the trajectory IDs. The graph is fully connected so that we can compare all trajectories to all trajectories. We place a similarity score on the edges of this graph by computing the Jaccard index of the trajectories the edge connects. To compute the Jaccard index of two trajectories, we think of them as sets of event codes. For an edge *T*_1_ → *T*_2_, the Jaccard index is then the ratio of the number of event codes in the intersection of *T*_1_ and *T*_2_ versus the number of event codes unique to *T*_1_ and *T*_2_. This is the graph we cluster with MCL (or another clustering algorithm). The cluster of a trajectory is then the cluster to which its trajectory ID (i.e. graph node) is assigned. The advantage of this clustering strategy is that all trajectories are guaranteed to be assigned to a single cluster.

#### TriNetX Dataworks data set for bladder cancer patients

The data used in this study was collected in December 2021 from the TriNetX Dataworks Network [19], which provided access to electronic medical records (diagnoses, procedures, medications, laboratory values, genomic information) from approximately 110,627,025 patients from 74 healthcare organizations. TriNetX, LLC is compliant with the Health Insurance Portability and Accountability Act (HIPAA), the US federal law which protects the privacy and security of healthcare data, and any additional data privacy regulations applicable to the contributing HCO. TriNetX is certified to the ISO 27001:2013 standard and maintains an Information Security Management System (ISMS) to ensure the protection of the healthcare data it has access to and to meet the requirements of the HIPAA Security Rule. Any data displayed on the TriNetX Platform in aggregate form, or any patient level data provided in a data set generated by the TriNetX Platform, only contains de-identified data as per the de-identification standard defined in Section §164.514(a) of the HIPAA Privacy Rule. The process by which the data is de-identified is attested to through a formal determination by a qualified expert as defined in Section §164.514(b)(1) of the HIPAA Privacy Rule. Because this study used only de-identified patient records and did not involve the collection, use, or transmittal of individually identifiable data, this study was exempted from Institutional Review Board approval.

We extracted a cohort of bladder cancer patients from TriNetX Dataworks [19]. Bladder cancer is one of the main driver cases for Project ATHENA (Augmenting Therapeutic Effectiveness through Novel Analytics) [20] we participate in. Bladder cancer has multiple stages and bladder cancer patients have a high risk of disease recurrence and progression to different stages. Hence studying bladder cancer trajectories is useful. To the best of our knowledge, bladder cancer trajectories extracted from medical event histories have not yet been studied in related work.

We identify bladder cancer patients as patients who have a record of a diagnosis “Malignant neoplasm of bladder” (ICD10: C67 or ICD9: 188) or a diagnosis “Personal history of malignant neoplasm of bladder” (ICD10: Z85.51 or ICD9: V10.52). We extracted this bladder cancer cohort in December 2021 and this gave us a data set with around 128 thousand patients and around 47 million timestamped ICD9/ICD10 diagnosis codes for those patients. As a point of comparison, for example, the data set used by Jensen et al [1] has around 6.2 million patients and 101 million diagnosis codes. Hence our data set has fewer patients, but far denser diagnosis histories. In addition to ICD codes, the TriNetX Dataworks data set also contains data for bladder cancer-specific treatments such as MVAC chemotherapy, radical cystectomy, and intravesical therapy. We assign these treatments unique medical event codes so they can be used in addition to the ICD codes as inputs to PTRA.

#### The PTRA tool

While PTRA is designed as a library for implementing analyses of medical event histories, it also implements a concrete use case. A command-line interface (CLI) is implemented to use PTRA as a tool for analyzing data extracted from the TriNetX Dataworks database [19]. It is useful to go over this CLI because it can help to understand the experiments we set up to evaluate PTRA.

The PTRA CLI for analyzing TriNetX is shown below in Listing 6:

ptra patientInfoFile diagnosisInfoFile diagnosesFile outputPath

 —nofAgeGroups nr —minPatients nr

 —maxYears nr —minYears nr

 —maxTrajectoryLength nr —minTrajectoryLength nr

 —iter nr —lvl nr

 —pfilters [age70+|age70-|male|female|NMIBC|MIBC|mUC|…]

 —tfilters neoplasm|bc

**Listing 6**. PTRA CLI tool for analyzing TriNetX data.

The CLI lists 4 required parameters: 1) a CSV input file with patient information such as sex, age, etc; 2) a diagnosis info file that maps diagnosis codes onto medical descriptors: e.g. XML file with ICD10 hierarchy from the WHO [10] or a CSV with CCSR categorization of the ICD10 hierarchy [11]; 3) a CSV file with timestamped ICD10/ICD9 diagnoses for the patients; 4) a path where to write the output.

There are optional parameters to further steer the trajectory building. The patient sampling for calculating RR scores compares patients similar in terms of age and sex. The age range for two patients to be considered similarly aged can be configured (--nofAgeGroups). The minimum number of patients in a trajectory can be set. The maximum and minimum time between diagnoses can be set, as can the maximum and minimum length of a trajectory. The number of iterations for the sampling process can be set (--iter). The level of the ICD diagnosis codes within the ICD hierarchy can be set so that ICD codes may be combined for analysis. Finally, there are filter options. A first set of filters (--pfilters) can be used to restrict the patients for which trajectories are calculated: e.g. only people above 70 years old, only females, only patients who are in the non muscle-invasive bladder cancer (NMIBC) stage, etc. Then there is a second filter option (--tfilters) that can be used to reduce the trajectory output, e.g. to include only trajectories with a cancer-related output.

There are additional options that are not listed in listing 6. We refer to the README with comprehensive documentation on our github repository for more details [17]. The PTRA tool is distributed as a single executable for Linux and can be compiled from source or downloaded directly from the repository.

**Fig 1:**
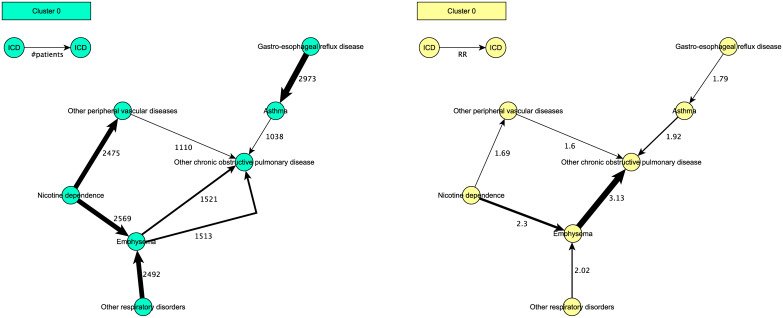
Example trajectory cluster extracted from TriNetX Dataworks (bladder cancer population). The graph on the left is annotated with the number of patients for consecutive diagnoses in a trajectory. The graph on the right represents the same trajectory cluster, but it is annotated with the RRs for each diagnosis pair instead. The graph on the left has more edges than the graph on the right because it shows all distinct trajectories annotated with their patient numbers. For example, different trajectories end up in “Other chronic obstructive pulmonary disorder” while having different starting points: “Nicotine dependence” or “Other respiratory disorders”.

A PTRA run creates multiple output files:

1. a .tab file with the selected diagnosis pairs and their relative risk scores.
2. a .tab file with the trajectories found.
3. a folder with clustered trajectory output. This folder contains up to four files:
  (a) a .csv file with cluster information linking patient, trajectory, and cluster.
  (b) a .csv file linking patient to TriNetX patient identifier and selected or derived TriNetX information about that patient (sex and age at event of interest). The two .csv files can be used to plot statistics per cluster (Julia script included in PTRA source code).
  (c) two graph modeling language (.gml) files with clustered trajectories encoded as a graph per cluster. The .gml format is a standardized format for storing graphs and such files can be visualized with external tools such as yED. There is one .gml file where trajectory transitions are annotated with the number of patients in the trajectory so far, and a second .gml file where the trajectory transitions are annotated with the relative risk score for the diagnosis pairs. An example is shown in Fig 1.

For more details on the outputs generated by PTRA, we refer to our README on github [17].

#### PTRA cluster interpretation

An example output for clustering trajectories with PTRA is shown in Fig 1. It shows the same cluster twice: once annotated with the number of patients and once annotated with the relative risk scores between diagnoses. The thickness of the connecting lines increases with the number of patients or relative risk scores respectively. The cluster shows that there are multiple trajectories leading to specific diagnoses. For example, there is a trajectory from “Nicotine dependence” to “Emphysema” to “Other chronic obstructive pulmonary disease”, but also a trajectory from “Gastro-esophageal reflux disease” to “Asthma” to “Other chronic obstructive pulmonary disease”. The first trajectory shows the negative impact of smoking, whereas the second trajectory reveals that asthma is often first misdiagnosed as gastro-esophageal reflux disease.

In order to interpret a PTRA cluster, there are a number of steps we recommend:

1. Identify the *key diagnoses* in a cluster. These are the diagnoses with the most connections in the graphical output. For example, in Fig 1 these are “Other chronic obstructive pulmonary disease” and “Emphysema”.
2. Identify the *common trajectories* by following the arrows that lead up to or start from key diagnoses.
3. Identify the *diagnoses with the highest risk* to predate or follow a (key) diagnosis. These can be spotted by looking at the thickest lines in the graphical output. For example, in Fig 1, the highest risk score is found for the transition of “Emphysema” to “Other chronic obstructive pulmonary disease”. It shows that patients diagnosed with “Emphysema” have three times more chance to be afterwards diagnosed with “Other chronic obstructive pulmonary disease” than patients that have not been diagnosed with “Emphysema”.
4. Check the *number of patients* from one diagnosis to another and compare it to the relative risk score. For example, in Fig 1, we see the highest number of patients between “Gastro-esophageal reflux disease” and “Asthma”. In comparison, the relative risk score is not as high as for other diagnoses. This suggests there are many patients diagnosed with “Asthma” that are not diagnosed with “Gastro-esophageal reflux disease”. In this case, this hints that the latter may be a misdiagnosis for the former.

#### The PTRA framework

The PTRA package is organized as a framework that defines APIs to implement trajectory analyses for medical event histories. We discuss code organization, how to add data filters, and the protocols for implementing a new use case in S1 Appendix.

### Benchmarks

To validate the PTRA output and its computational performance, we benchmark the PTRA tool with different parameters on the bladder cancer cohort we extracted from the TriNetX Dataworks data set.

#### Experiments

We set up different runs with PTRA to analyze the bladder cancer cohort we extracted from TriNetX Dataworks. As a point of reference, we set up a run that extracts all trajectories for the full cohort. This is easiest to compare to the experiments from the papers on the original methods which focus on processing complete diagnosis histories of all patients in a population [1, 2, 8].

We additionally set up an experiment that extracts bladder cancer-specific trajectories. For this experiment, we use PTRA-specific features. Concretely, we restrict the output to return trajectories that contain at least one bladder cancer-related diagnosis. This comprises ICD10 codes C67, C77, C78, C79, as well as medical procedures such as radical cystectomy, MVAC chemotherapy, and intravesical therapy that are registered separately in TriNetX Dataworks. The PTRA algorithm automatically maps these procedures onto unique medical event codes for this purpose. Additionally, we set up PTRA to remap the ICD codes onto CCSR categories for assisting medical interpretation of the trajectories.

Table 1 gives an overview of the parameter settings for the different runs. For overlapping parameters, we chose similar values as those used in related work [1–6, 8].

**Table 1:** Experiment parameters for runs generating 1) all trajectories based on full ICD histories and 2) bladder cancer-specific trajectories found using ICD histories and bladder cancer-specific treatments.

Parameter	General BC	BC specific
–nofAgeGroups	10	10
–minPatients	100	100
–maxYears	5Y	5Y
–minYears	6M	1D
–minTrajectoryLength	3	3
–maxTrajectoryLength	5	5
–lvl	2	2
–iter	400	400
–tfilters	none	bc
diagnosisInfoFile	ICD10	CCSR

#### Server and software versions

All of our experiments ran on a 36-core server, consisting of two 18-core Intel Xeon E5–2699v3 Haswell CPUs clocked at 2.3Ghz. The server is equipped with 256GB RAM and two 400GB SSD disks for storing local data. The machine runs Ubuntu 20.04.4 LTS Linux. PTRA was compiled using Go 1.18.4. For clustering, we used the MCL program version 11–294. The trajectory output of PTRA (in .gml format) was visualized using yEd 3.21.1. Plotting and additional analysis was done with Julia 1.7.3.

## Results

### Validation hypothesis generation

We next discuss the general and bladder cancer-specific trajectories found for the bladder cancer cohort extracted from TriNetX Dataworks. Plots are listed in S2 and S3 Appendices.

#### General disease development patterns in bladder cancer patients in TriNetX Dataworks

In our first experiment, we generate trajectory clusters for the full cohort of bladder cancer (BC) patients. This means that we calculate trajectories using the full diagnosis histories of all patients in the cohort and collect all trajectories found this way. This is similar to the approach taken in related work [1, 2, 8].

This approach reveals clusters of highly prevalent, well-known chronic diseases such as cardiovascular disease, diabetes, dorsalgia, etc, which co-occur in an aging population such as BC patients. These clusters are not necessarily related to BC and this makes it difficult to zoom in on BC-specific development patterns. Nonetheless, we can assign medically valid interpretations to these clusters, showing the PTRA algorithm produces sensible results.

For the full BC cohort, PTRA finds 17 clusters (S2 Appendix). The 5 largest clusters found, have the following central diagnoses: heart disease (cluster 0), soft tissue disorder (cluster 1), musculoskeletal disorder (cluster 3), cardiorenal disease (cluster 4), and anxiety/sleep disorder (cluster 5). When examining age and sex-related characteristics per cluster, we see they confirm some well-known disease characteristics:

- the percentage of males is higher in the heart failure cluster.
- the percentage of females is higher in the clusters that involve anxiety disorder.
- the mean age of diagnosis of bladder cancer is similar across all clusters: on average 70 years of age.

While the clusters are clinically correct, the specific findings do not represent new knowledge per se, as the most general clusters of diseases tend to dominate in the output. A lot of clusters are also dominated by overarching and unspecific ICD codes such as “Other and unspecified soft tissue disorder, not elsewhere classified” and “Other joint disorders”. Additionally, no cluster includes BC as a diagnosis event. This is logical, as each patient is diagnosed with BC in the input data. Therefore the algorithm cannot sample comparison groups without BC (cf. Listing 3) and the statistics cannot pick it up as an event of interest. This makes it difficult to study BC-specific development patterns.

In our second experiment, we show how PTRA-specific features make it easier to study BC-specific trajectories. Specifically:

- We make use of CCSR categorization of ICD10 codes. The Clinical Classifications Software Refined (CCSR) aggregates ICD10 codes into clinically meaningful categories. This bypasses the problem of clusters being dominated by overarching or unspecific diagnoses.
- We include BC-specific treatments to the diagnosis histories for trajectory building. This gives a handle on identifying BC-specific trajectories. We also changed the minimum time between diagnoses from six months to one day to capture the fact that BC treatments may follow each other quickly.
- We use the trajectory filter mechanism to restrict trajectory output to trajectories that include at least one known bladder cancer-related diagnosis (ICD code) or treatment. This steers the PTRA algorithm to cluster and output only trajectories related to BC.

#### Bladder cancer development patterns in TriNetX Dataworks

Approximately 75% of patients with BC present in the early stage (non-muscle-invasive bladder cancer—NMIBC) of the disease, meaning that the disease is confined to the mucosa (stage Ta, carcinoma in situ [CIS]) or submucosa (stage T1). In this group, recurrence and progression remain problematic. Despite intensive treatment and follow-up schedule, these patients have an elevated risk for disease recurrence and a moderate to elevated risk for disease progression of up to 35–55% at a year follow-up [21]. Management of NMIBC consists of TURBT (operative removal of tumor) and bladder instillations with BCG (Bacillus Calmette-Guérin) plus intensive follow-up and maintenance BCG therapy. The rate of BCG non-responders amounts to 40%. This explains why the European Association of Urology states in their most recent guidelines that radical cystectomy should be offered to the very high-risk patients, even in early stages of the disease [22]. Removal of the bladder drastically diminishes the patients? quality of life, making it incredibly important to optimally discriminate between high- and very high-risk patients. Additionally, beforehand determination of response to treatment with intravesical BCG instillations in BC would increase the survival of patients while eliminating time loss and unnecessary use of medications. Management of late-stage (muscle-invasive bladder cancer—MIBC) disease consists of radical cystectomy with or without chemotherapy, depending on eligibility of the patient for surgery/chemotherapy.

We need to be able to better stratify BC patients. In this perspective, there is a need to:

1. Identify BC patients who are at the highest risk for disease progression.
2. Predict treatment response.

In pursuit of these goals, we analyze the PTRA trajectories found for the BC patients in the TriNetX Dataworks database. We use the PTRA trajectory filters to only output and cluster trajectories that contain a BC-related diagnosis or treatment.

**Fig 2:**
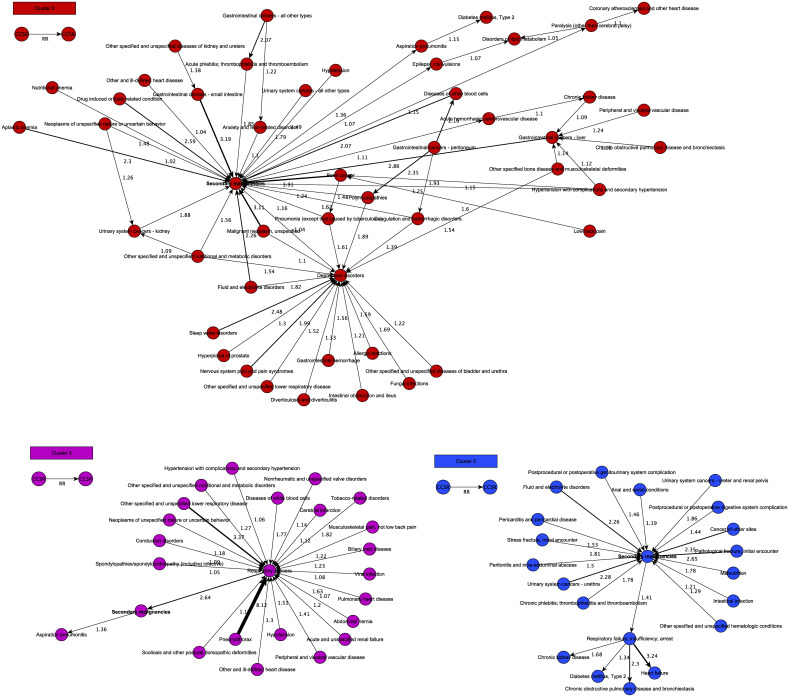
Clusters for secondary malignancy. PTRA clusters with secondary malignancy as central diagnosis (in bold). The clusters show different trajectories leading up to secondary malignancy. For example, cluster 0 shows trajectories where hematological and drug-induced disturbances precede secondary malignancy, whereas cluster 5 shows infection-related events. The clusters suggest a different development of events after the secondary malignancy diagnosis: cerebrovascular disease, epilepsy, and paralysis for cluster 0, and respiratory insufficiency for cluster 5.

**Fig 3:**
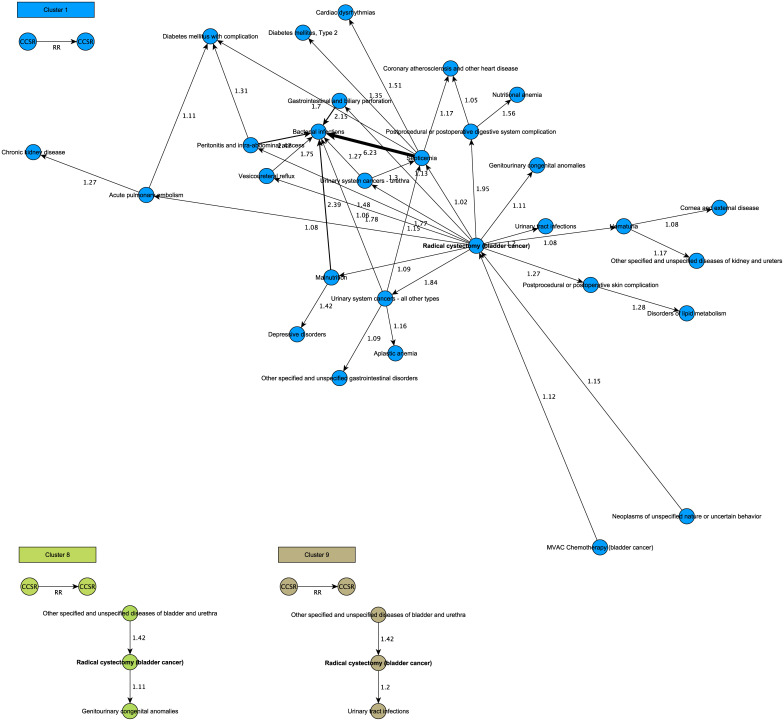
Clusters for radical cystectomy. PTRA clusters showing potential complications after radical cystectomy (in bold). Ranked from highest to lowest risk, this includes: post-procedural or postoperative digestive system complication, reflux disease, peritonitis and intra-abdominal abscess, postoperative skin complication, urinary tract infection and gastrointestinal and biliary perforation. Also rare, but severe complications such as septicemia and pulmonary embolism are identified, albeit with lower relative risk scores than the other complications. These findings seem to correlate with complications of radical cystectomy described in literature.

**Fig 4:**
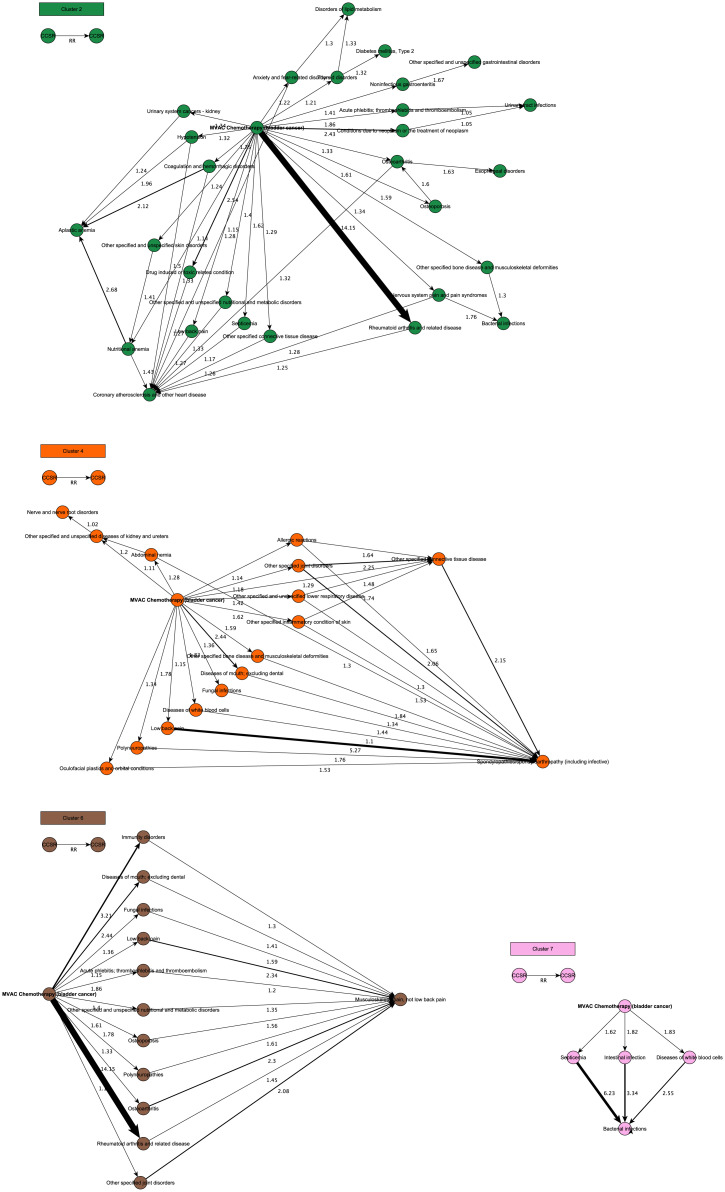
Clusters for MVAC chemotherapy and effects. PTRA clusters with MVAC chemotherapy as a central event (in bold). The clusters show follow-up events that are known side-effects described in literature such as: drug-induced toxicity, acute phlebitis, thromobphlebitis, and thromboembolism, fungal infections, and polyneuropathy. However, the clusters also highlight a high risk of rheumatoid arthritis with a relative risk score of 14.15, which is a remarkable finding as flare up of auto-immune diseases with MVAC chemotherapy is not assumed to be conventional.

Using PTRA, 10 clusters of disease trajectories were discovered. The dominant diagnostic event in each of the clusters is secondary malignancy (cluster 0, 3 [respiratory cancer] and 5) (Fig 2), radical cystectomy (cluster 1, 8 and 9) (Fig 3), and MVAC (Methotrexate-Vinblastine-Doxorubicin [Adriamycin]-Cisplatin) chemotherapy (cluster 2, 4, 6 and 7) (Fig 4).

Cluster 0, 3 and 5 show events preceding the diagnosis of secondary malignancy, presenting risk factors or biomarkers for detecting secondary malignancy (Fig 2). Multiple types of hematological deviations are identified as preceding events in cluster 0: aplastic anemia having the highest risk ratio (RR) (2,3), followed by fluid/electrolyte disturbances (2,26), diseases of white blood cells (2,07) and nutritional anemia (1,48). This cluster also revealed drug-induced or toxicity-related conditions preceding secondary malignancy with a relatively high RR (2,59). This phenomenon seems logical because the need for terminating treatment due to side effects diminishes the chances of treatment success, which in turn could lead to disease progression. Cancer of the small intestine is the event preceding secondary malignancy with the highest RR (3,19). This finding is surprising because the small intestine is not the primary location for metastasis and primary cancer of small intestine itself is a very rare event. Most often BC will spread to lymph nodes, bones, lung, liver, and peritoneum [23, 24]. Due to the nature of the dataset that is used (TriNetX), we lack granularity in the terminology to clearly explain the underlying disease physiology of these findings. We require more detailed information on the definitions of used terms. However, the dataset used to develop this algorithm does not contain this detailed amount of information. More research exploring this algorithm on different datasets is needed.

When comparing cluster 0 and cluster 5, both having secondary malignancy as central diagnosis, different types of trajectories are revealed. Within cluster 0, multiple preceding events are hematological and drug-induced disturbances while cluster 5 shows more infection-related events (pericarditis, intestinal infection, peritonitis and intra-abdominal abscess etc.). This seems to have an influence on events occurring after diagnosis of secondary malignancy; cluster 0 leading to cerebrovascular disease, epilepsy and paralysis and cluster 5 leading to respiratory insufficiency.

Cluster 1, 8 and 9 reveal potential complications of radical cystectomy (Fig 3). When ranked from most to least likely: post-procedural or postoperative digestive system complication (RR 1,95), reflux disease (RR 1,78), peritonitis and intra-abdominal abscess (RR 1,75), post-procedural or postoperative skin complication (RR 1,27), urinary tract infection (RR 1,2) and gastrointestinal and biliary perforation (RR 1,13). Severe complications such as septicemia and pulmonary embolism are also identified, though rare compared to the other events (RR 1,02 and 1,08). These seem to correlate with complications described in literature.

Cluster 2, 4 and 6 reveal MVAC chemotherapy and its effects (Fig 4). The clusters show that rheumatoid arthritis (RA) and related disease is one of the most common events after MVAC therapy (with RR of 14,15), along with diseases of the mouth (RR 2,44), drug-induced toxicity (RR 2,54), acute phlebitis, thrombophlebitis, and thromboembolism (RR 1,86) and others such as, fungal infections (RR 1.36), polyneuropathy (RR 1,78) etc. Most of these events are known side-effects described in literature [25]. However, the high risk of RA after MVAC is noteworthy. Cancer and autoimmune disease are closely related, and many therapeutic antibodies are widely used in clinics for the treatment of both diseases. At the same time, certain immunotherapies used in cancer treatment can induce autoimmune reaction [26]. With MVAC chemotherapy, flare up of auto-immune diseases is not conventional. Specifically, because Methotrexate is used in low doses for treating RA. A clinical correlation between the RA and MVAC does exist, the order of events in our trajectories, however, is remarkable.

**Fig 5:**
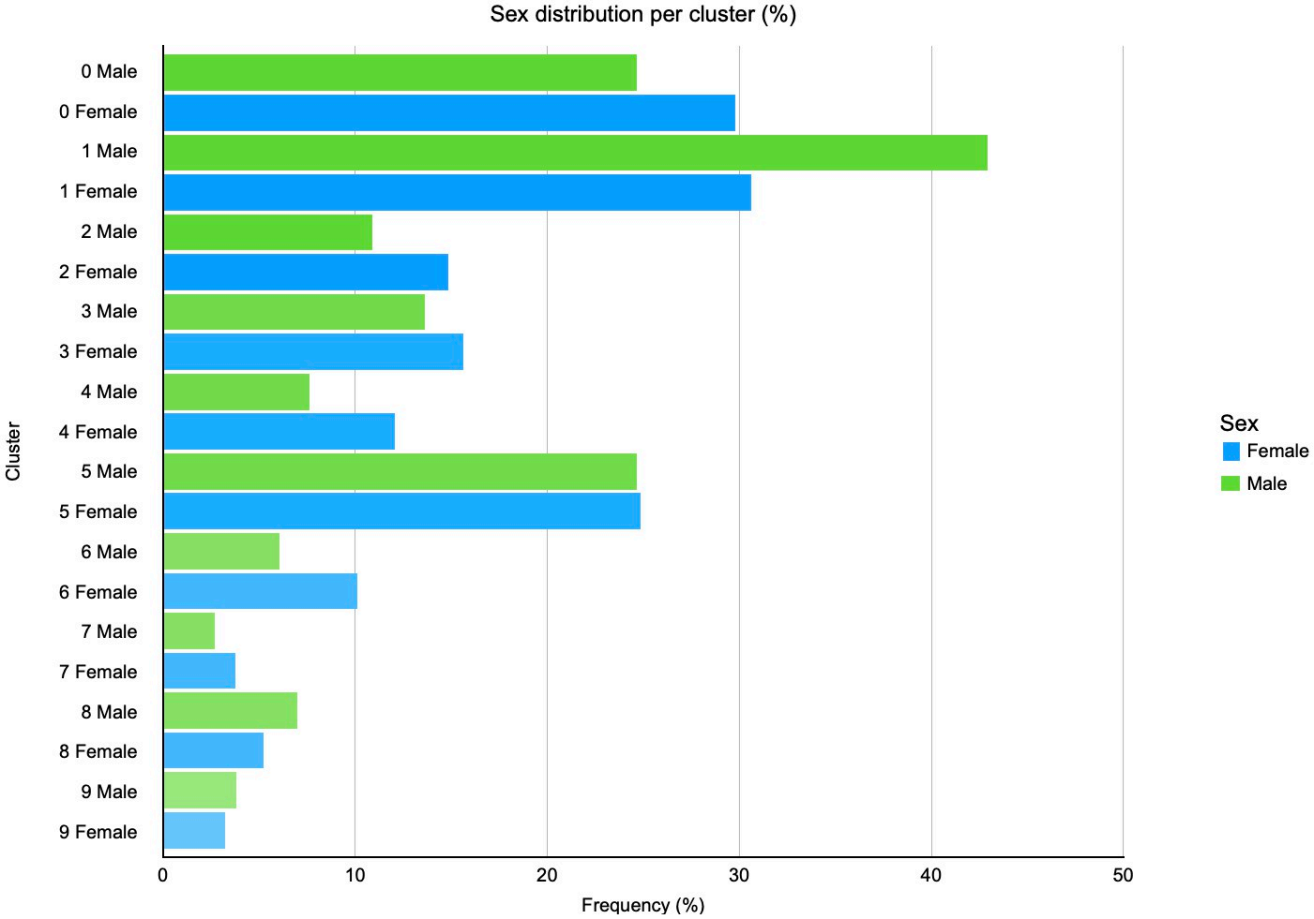
Gender distribution per cluster. This graph shows for each PTRA cluster the percentage of female patients versus the percentage of male patients that fall into that cluster. It shows, for example, for the MVAC clusters (clusters 2,4,6 from Fig 4), that the percentage of females is higher than the percentage of males. In contrast, the clusters with radical cystectomy as central event (clusters 1, 8, 9 from Fig 3), contain more males than females. This is consistent with literature that reports female gender as a risk factor for disease recurrence, progression, and cancer-specific mortality requiring more advanced treatments.

Using PTRA, we can also analyze the patient characteristics of each of our clusters (Fig 5). Gender analysis shows that in clusters with MVAC as dominant diagnostic event, the percentage of females is higher, while clusters with radical cystectomy are male dominated. This is consistent with earlier studies, where female gender is reported as a risk factor for disease recurrence, progression, and cancer-specific mortality requiring more advanced treatments [27]. The literature suggests that female gender in patients diagnosed with BC is associated –though inconsistently– with higher stage at presentation and poorer outcomes.

**Fig 6:**
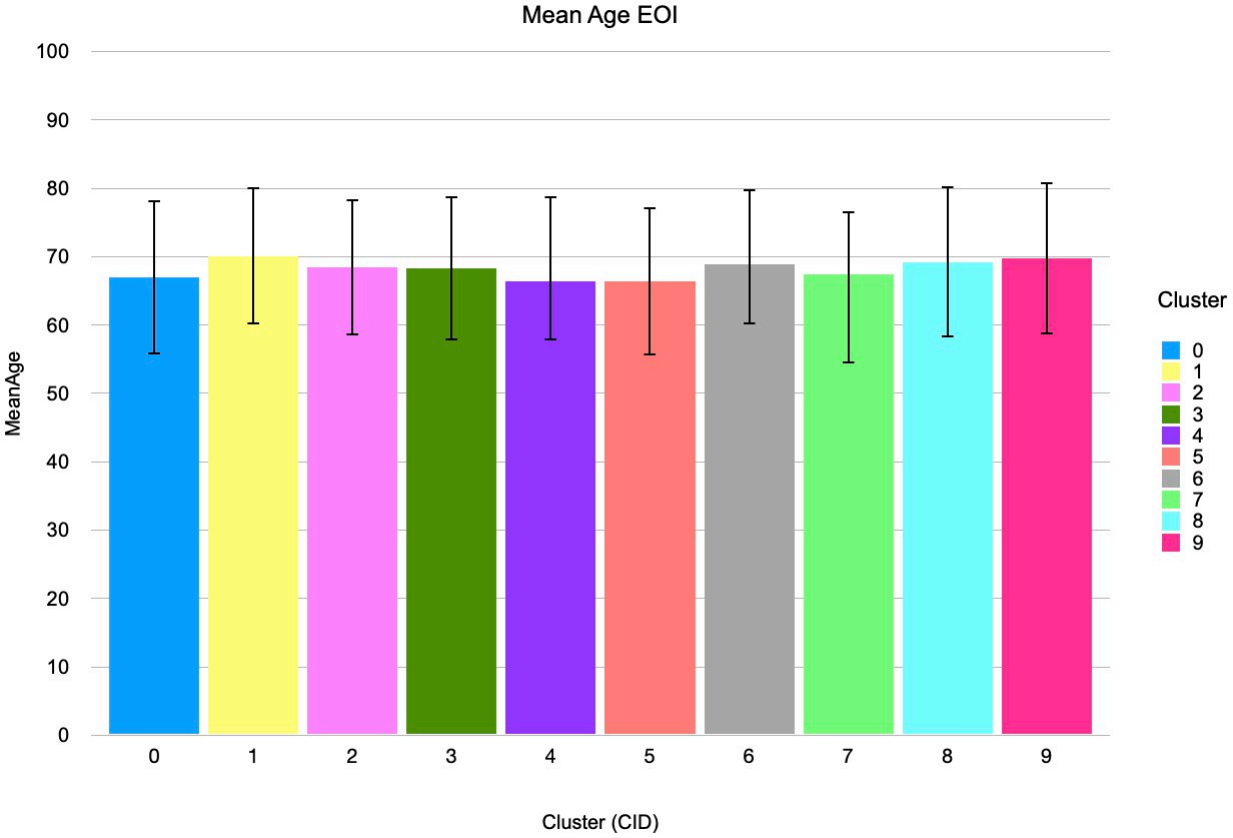
Average age of onset of BC per cluster. This graph shows fro each PTRA cluster the average age a patient in that cluster is diagnoses with bladder cancer. It shows for the MVAC clusters (clusters 2, 4, 6 from Fig 4) that the average age of onset of disease is lower compared to other clusters: +/-63 years vs +/-70 years. We assume this is because toxicity is a major concern with MVAC chemotherapy, especially in an elderly population with multiple comorbidities. Therefore older patients are often ineligible for MVCA therapy.

Age analysis in Fig 6 reveals that in clusters were MVAC chemotherapy is a dominant diagnosis event, the average age of onset of disease is lower compared to other clusters (+/-63 years vs +/-70 years). In contrast, older adults have a higher mortality due to BC than younger individuals. We would therefore expect our older population of BC patients to have progressive disease and need additional treatment with surgery or chemotherapy. However, toxicity is a major concern with MVAC chemotherapy, particularly in an elderly population with multiple comorbidities. Therefore, an older population is more often ineligible for MVAC therapy. This explains why the mean age in these clusters is lower.

#### Limitations

While working with ICD-10 codes can provide valuable information about patient trajectories, there are several limitations we need to address:

- Coding errors and inconsistencies: Assigning ICD-10 codes can be a complex task, and errors or inconsistencies may occur during the coding process. These errors can lead to inaccuracies in patient trajectory analysis, potentially impacting the interpretation of data. An illustrative example is the diagnosis of sepsis. Sepsis, a life-threatening blood infection, is typically diagnosed based on blood cultures. Given the urgent need for immediate intervention in sepsis cases, the coding aspect is sometimes overlooked and not added to the electronic medical records retrospectively [28]. Another observation from our experiments highlighted the inconsistencies of disease progression diagnoses. We noticed instances where patients were receiving therapy for advanced-stage cancer, while their records still indicated an early-stage cancer code. The presence of coding inconsistencies, as exemplified by this discrepancy, can result in substantial information loss, underscoring the importance of addressing this limitation.
- Lack of granularity: ICD-10 codes provide a standardized system for classifying diseases and health conditions, but they often lack the level of detail necessary to capture the complexity of patient trajectories. The codes may not fully capture the nuances of a patient’s specific condition, variations in disease severity, or the progression of symptoms over time. In our experiment, we encountered this limitation when it came to diagnosing secondary malignancy. This diagnosis can be interpreted in different ways, potentially indicating the development of a new primary cancer or it can indicate the progression of an existing disease with the occurrence of metastases. Due to the lack of more detailed information, we were unable to draw any definitive conclusions.

To overcome the limitations associated with ICD-10 codes and the inconsistencies we encountered, it is crucial to augment the analysis with additional data sources and clinical information. This approach will ensure a more comprehensive understanding of patients’ healthcare journeys. In future work, the following steps can be taken to address specific inconsistencies:

- Sepsis case: Incorporate supplementary data such as lab results and the diagnosis of bacteria in blood cultures to aid in assigning the sepsis diagnosis. Additionally, consider including information on antibiotics administered as treatment for sepsis. By integrating these data points, a more accurate and comprehensive picture of the sepsis diagnosis can be obtained.
- Metastasis case: Implement an automated system that adjusts the disease stage (early/late stage cancer) once a treatment for late-stage cancer is recorded. This would ensure that the coding accurately reflects the patient’s current disease status and progression. By incorporating these strategies and leveraging additional data sources, researchers and healthcare professionals can mitigate the limitations associated with ICD-10 codes and gain a more holistic understanding of patients’ healthcare journeys.

### Computational benchmarks

The computational costs for our experiments are shown in Table 2. The table shows the runtimes and RAM use, as well as the number of trajectories and clusters found for each experiment. As we discussed in Methods section, all benchmarks are run on a single multicore server. The difference in runtimes is because the run that outputs bladder cancer-specific trajectories places restrictions on which trajectories the PTRA algorithm attempts to complete. For example, each trajectory has to have at lease one cancer-related ICD code or treatment, eliminating a large set of trajectories, which are consequently never clustered, reducing the time spent on clustering and outputting, etc.

**Table 2:** Benchmark results comparing 1) a run searching for all possible disease trajectories using only ICD data from the bladder cancer cohort versus 2) a run searching for bladder cancer-specific trajectories using both ICD data and bladder cancer treatment data.

Run	Time	RAM	Trajectories	Clusters
General BC	19h 14m	10GB	775	17
BC specific	9h 24m	10GB	259	10

The runtimes shown in Table 2 are in an acceptable range for most researchers, as the infrastructure we use to run the benchmarks can be rented in the cloud for a few dollars per hour: e.g. m5.16xlarge for 3.07$ per hour on AWS (pricing September 2022). This computational efficiency is one of the contributions of PTRA.

**Fig 7:**
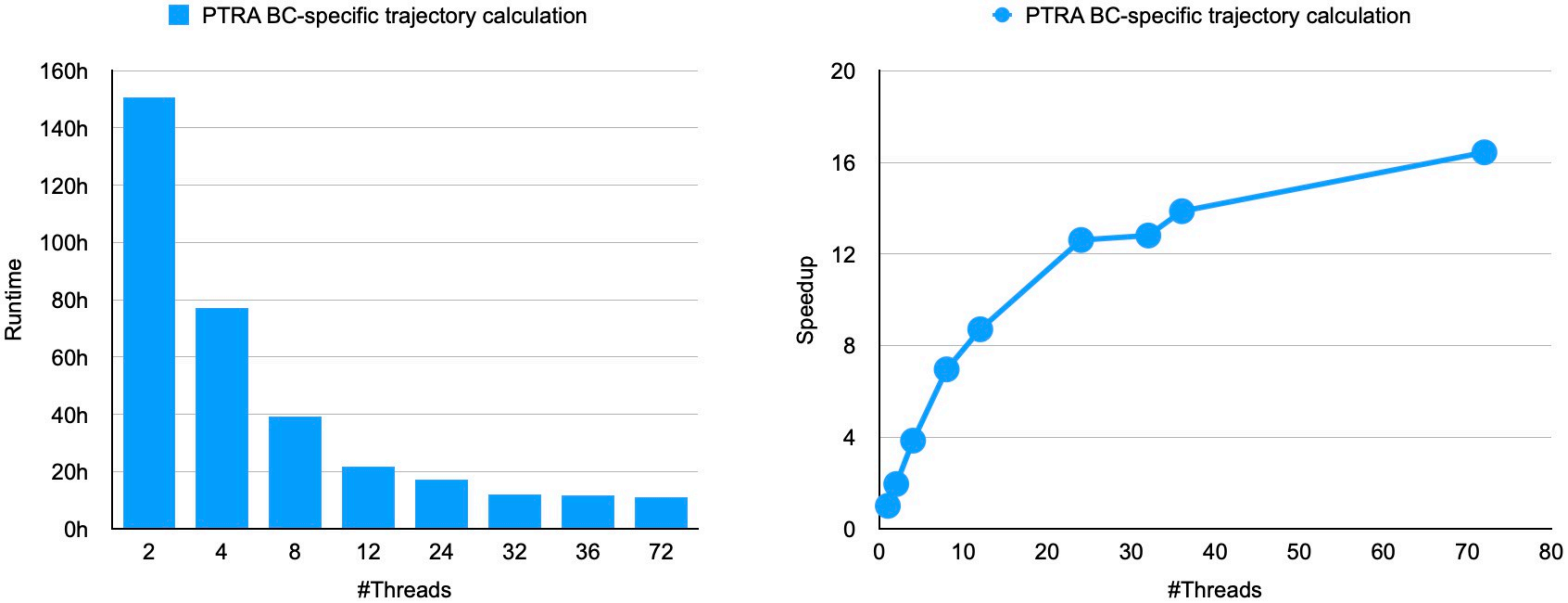
Strong scaling experiment. This graph shows the runtime of the BC specific workload for 1 to 72 threads on our 36-core Intel Xeon server with hyper threading. While linear speedup tops off around 12 threads, we see a reduction in overall runtime from around 150 hours on 1 thread to around 9 hours at maximum number of threads. This speedup is thanks to the parallelization of the RR calculation and the trajectory building.

In Fig 7 we additionally show a strong scaling experiment for PTRA. We executed our BC specific workload on our 36-core Intel Xeon server with hyper threading by limiting the number of hyper threads to be used from 1 to 72. In theory, such a server can achieve a linear speedup of 36x for a parallel workload without sequential sections. In PTRA, the RR calculation and the trajectory building are parallelized. The graph shows linear speed up starts to top off around 12 threads. However, the overall reduction in runtime is from around 150 hours on 1 thread to around 9 hours using the full machine. This is a significant reduction in waiting time compared to what the expected single-threaded runtime would be. Note that in this benchmark we did not include the time spent in the clustering library we use, hence there is a 15 minute difference compared to the total runtime in Table 2 for the runtime on 72 threads.

We want to emphasize that the good computational runtimes of PTRA are not a trivial result. The original work by Jensen et al. [1] claims requiring complex cluster computing, but no source code was released. While Paik et al [7] released a source code for calculating trajectories, their method is much simplified compared to the method in PTRA and other related work. Specifically, they define the relative association (RA) metric to calculate a score for selecting diagnosis pairs that can be combined into trajectories. The RA calculates a score by counting the instances of co-occurrences of diseases in the population versus total occurrences of those diseases in the population. This is a much simpler calculation compared to calculating the relative risk score (RR) which is used in PTRA and related work. As we discussed, the calculation of the RR scores requires executing multiple statistical sampling experiments, which greatly adds to the computational complexity of PTRA, but it also has a different statistical significance.

## Conclusion

PTRA is a software package for exploring patient development by analyzing trajectories extracted from the medical event histories of a patient population. While based on the statistical method developed previously by Jensen et al [1], the PTRA algorithm introduces several modifications and extensions to implement a more practical tool. The PTRA algorithm is generalized to work on any type of timestamped medical event data, introduces a new clustering strategy, proposes filter mechanisms for restricting analysis to subpopulations or for restricting the trajectory output to events of interest, and uses parallelization to allow an efficient implementation that can execute on a single multicore server. As an experiment, we show how these features make it possible to analyze patient development for bladder cancer patient data extracted from the TriNetX Dataworks database. We show this experiment produces trajectory clusters with a sound medical interpretation. Computational benchmarks show runtimes and resource use on a single multi-core server that are easily affordable. PTRA is furthermore managed as an open source project where the code is organized as a framework so that researchers can reuse it to analyze new data sets. This makes PTRA a valuable tool for the research community.

In future work, we plan to improve the PTRA tool by developing a graphical user interface (GUI) that makes it possible to interactively view the trajectories. One option may be to combine PTRA with the improved visualization and user interface work by Paik et al [7] for a Jensen-like trajectory algorithm. We additionally think further improvements are possible in the clustering step, for example by trying different distance metrics in addition to the Jaccard Index such as for example an algorithm based on dynamic time warping [3, 29]. Another topic of interest is extending PTRA so the trajectories can be used as inputs to predictive models, similar to what is proposed in related work [4, 6, 8]. Additionally, we want to try to pool more data related to bladder cancer as inputs to PTRA to further study the development patterns in bladder cancer patients.

## Supporting information

S1 Appendix The PTRA framework. This appendix presents an overview of the PTRA implementation framework and how it can be extended to implement trajectory analysis on new data sets.
(PDF)

S2 Appendix Clusters found for the full bladder cancer cohort. This appendix lists all clusters found for the full bladder cancer cohort while generating all found trajectories.
(PDF)

S3 Appendix Bladder cancer-specific clusters. This appendix lists all bladder cancer-specific clusters found for the full bladder cancer cohort, using the TriNetX ICD diagnosis codes, as well as bladder cancer-specific treatments. The trajectories shown are filtered to only show trajectories where at least one medical event is related to cancer.
(PDF)
