# Supplementary material for: A software package for efficient patient trajectory analysis applied to analyzing bladder cancer development
Source: PLOS Digit Health. 2023 Nov 22;2(11):e0000384. doi: 10.1371/journal.pdig.0000384 (PMC10664923; doi:10.1371/journal.pdig.0000384)
Supplement: S1 Appendix — This appendix presents an overview of the PTRA implementation framework and how it can be extended to implement trajectory analysis on new data sets. (PDF) [file pdig.0000384.s001.pdf]

S1 Appendix: The PTRA framework

Charlotte Herzeel[1]

1 ExaScience Lab, imec, Leuven, Belgium

* Charlotte.Herzeel@imec.be

The PTRA framework

The PTRA package is organized as a framework that defines APIs to implement trajectory analyses for medical event histories. We discuss code organization, how to add data filters, and the protocols for implementing a new use case.

Code organization

The PTRA code is organized as follows:

- `ptra`: the top-level package containing the main program, README, license, and sub packages:
  - `trajectory`: this package contains the core of PTRA. It implements key algorithms and data structures for calculating relative risk ratios of event pairs and for building trajectories from those pairs. It also contains the interfaces and definitions of basic data filters.
  - `cluster`: this package contains the algorithms for clustering trajectories.
  - `plot`: this package contains the code for formatting and writing PTRA output to disk.
  - `app`: this package contains all code specific to use cases. This where parsing of input files into core data structures is located. It also contains use case-specific data filters. Finally, it is also where to place the use-case specific command-line interface.
  - `utils`: this package contains some utility data structures and functions.

Adding filters

The PTRA package defines a filter mechanism to reduce data input and output. Two types of filters are possible: patient filters and trajectory filters. The `trajectory` package defines a standard set of patient filters (`ptra/trajectory/filters.go`). The standard API to implement a patient filter is given by the interface shown in Listing 1.

```
type PatientFilter func(patient *Patient, info interface{}) bool
```

Listing 1. API for implementing a patient filter.

A patient filter is a function that takes as input a patient object and returns a boolean as output. When a filter returns `false`, the patient is removed from input before calculating trajectories. When instead the filter returns `true`, the patient is kept for trajectory calculation. As an example, consider the very simple filter for removing males shown in Listing 2.

```
func MaleFilter(p *Patient) bool {
    return p.Sex != Male
}
```

Listing 2. API for implementing a patient filter.

Patient filters are called by the function `ApplyPatientFilters` which is called during input parsing (e.g. `ParseTriNetxData` in `app/parseData.go`).

In order to implement a new patient filter, one has to implement the interface in Listing 1. The CLI also has to be extended to be able to pass the new filter: extend `getPatientFilter` in `ptra/main.go`.

To implement meaningful patient filters, one also has to have an understanding of the structure that implements patients, cf. `trajectory.Patient` and the functions that operate on this type. The most important fields of the struct are highlighted in Listing 3. Please consult the code documentation on github for details.

```
type Patient struct {
        PID, YOB, Sex, CohortAge int
        PIDString                string
        Diagnoses                []*Diagnosis
        EOIDate                  *DiagnosisDate
        DeathDate                *DiagnosisDate
}
```

Listing 3. Struct representation of a patient.

In addition to patient filters, it is also possible to implement trajectory filters, to control which trajectories are written to output. The standard API to implement a trajectory filter is shown in Listing 4. Analogous to patient filters, a trajectory filter is a boolean function that takes as input a trajectory and return `true` if the intent is to keep the trajectory for output or `false` if it is to be removed.

```
type TrajectoryFilter func(t *Trajectory) bool
```

Listing 4. API for implementing a trajectory filter.

An example trajectory filter is shown in Listing 5. It implements a filter that makes sure only trajectories containing a bladder cancer-related diagnosis are kept for output.

```
func BCFilter(exp *trajectory.Experiment) trajectory.TrajectoryFilter {
        return func(t *trajectory.Trajectory) bool {
                for _, did := range t.Diagnoses {
                        icdCode := exp.IdMap[did]
                        if len(icdCode) >= 3 {
                                subCode := icdCode[0:3]
                                if subCode == "C67" {
                                        return true
                                }
                        }
                }
                return false
        }
}
```

Listing 5. Example trajectory filter to remove trajectories without a bladder cancer-related diagnosis.

Trajectory filters are called by the function `BuildTrajectories` in `ptra/trajectory/trajectory.go`.

In addition to implementing the interface in Listing 4, implementing a new trajectory filter also requires extending the CLI so that filter can be passed: extend `getTrajectoryFilter` in `ptra/main.go`. Trajectory filters operate on trajectory objects implemented by the struct `trajectory.Trajectory`. It is crucial to look at the

fields of that type and the functions that operate on it (Listing 6). We refer to the source code documentation for details.

```
type Trajectory struct {
        Diagnoses       []int
        PatientNumbers  []int
        Patients        [][]*Patient
        ID              int
        Cluster         int
}
```

Listing 6. Struct representation of a trajectory.

Implementing a new use case

In order to implement trajectory analysis for a new data set, a five step protocol can be followed:

1. Parse the input data into a `trajectory.Experiment` structure.
2. Initialize the experiment's relative risk ratios.
3. Build the experiment's trajectories.
4. Output the found trajectories to disk.
5. Cluster the found trajectories and write the output to disk.

These steps are explained in the next sections.

1. Parse the data inputs

The core data structure for implementing trajectory analysis is the `trajectory.Experiment` structure shown in Listing 7:

```
type Experiment struct {
        Name                                        string
        NofAgeGroups, Level, NofDiagnosisCodes      int
        DxDRR                                       [][]float64
        DxDPatients                                 [][][]*Patient
        NameMap                                     map[int]string
        IdMap                                       map[int]string
        Cohorts                                     []*Cohort
        DPatients                                   [][]*Patient
        Pairs                                       []*Pair
        Trajectories                                []*Trajectory
}
```

Listing 7. Struct representation of a trajectory.

Some of these slots are initialised by ptra functions in subsequent steps of the protocol, but the following need to be initialized explicitly:

- `Name`: a name for the experiment. This name is used for creating file names when writing output to disk.
- `NofAgeGroups`: the number of age groups for the experiment. This can be a parameter passed via the CLI as in the TriNetX case cf. `ptra/main.go`.
- `Level`: this is an optional integer slot to hold a 'level'. It is only used for logging. For the TriNetX case, this refers to the level in the ICD10 hierarchy the analysis operates on.

- `NofDiagnosisCodes`: this is the number of diagnosis codes used in the input data. This number is used to size different data structures that are initialized for calculating RR scores. For example, the idea is that diagnosis codes/medical events are mapped on a unique analysis ID, counting from `0` to `NofDiagnosisCodes`. We can then initialize, for example, an array of size `NofDiagnosisCodes` and use the analysis ID of a diagnosis as an index into this array.

  For example, assume the following medical events occur in the diagnosis histories:

  ```
  Cough, Dyspnea, COPD, BMI>30, High Blood Pressure, Diabetes
  0      1        2     3       4                    5
  ```

  There are 6 medical events, so `NofDiagnosisCodes = 6`. We could assign them analysis IDs as shown by using a simple counter.

- `DxDRR`: this is a matrix that stores for each diagnosis pair the relative risk score for that pair. This matrix must be initialized when creating a `trajectory.Experiment` via the function `trajectory.MakeDxDRR`. That function's signature is: `func MakeDxDRR(size int) [][]float64`. It takes one size parameter, cf. the number of diagnosis codes in the input (`NofDiagnosisCodes`). The actual RR are calculated and filled in at a later step of the protocol (step 2).

- `DxDPatients`: this is a matrix that stores for each diagnosis pair the patients diagnoses with that pair. The matrix must be initialized when creating a `trajectory.Experiment` via the function `trajectory.MakeDxDPatients`. This function takes a single size parameter, cf. `NofDiagnosisCodes`.

- `NameMap`: maps analysis IDs for diagnoses onto a medical event name. For example: `0 --> Cough, 1 --> Dyspnea, 2 --> COPD, 3 --> BMI>30, 4 --> High Blood Pressure, 5 --> Diabetes`. This map should be extracted from input data. For example, in the TriNetX case, this map is extracted from the ICD10 hierarchy definition, cf. the function `initializeIcd10AnalysisMapsFromXML`.

- `IdMap`: maps analysis IDs for diagnoses onto diagnosis ID that occurs in the input. For example, in the TriNetX case, this would be ICD10 code of a diagnosis. See `initializeIcd10AnalysisMapsFromXML` as an example.

- `Cohorts`: this represents an array with all cohorts in the experiment. The statistical model behind the RR calculation divides the patients into cohorts according to their sex and age. This is to make sure statistical sampling experiments can compare similar patients (to avoid Simpson's paradox). Concretely, patients are split into male and female patients. Both sexes are subsequently split up into age groups.

  For example:

  `Males: cohort1: age = [0-30[, cohort2: age = [30-60[, cohort3: age = [60-90[, cohort4: age = [90-120]`

  `Females: cohort5: age = [0-30[, cohort6: age = [30-60[, cohort7: age = [60-90[, cohort8: age = [90-120]`

  Cohorts are initialized by calling the function `trajectory.InitializeCohorts`. The signature of this function is: `func InitializeCohorts(ps *PatientMap, nofAgegroups, nofDiagnosisCodes int) []*Cohort`

– The `ps` argument represents the patient object parsed from the input. The patients should be passed as a `trajectory.PatientMap` object:

```
type PatientMap struct {
        PIDStringMap map[string]int
        Ctr          int
        PIDMap       map[int]*Patient
        MaleCtr      int
        FemaleCtr    int
}
```

Listing 8. Struct representation of a trajectory.

The slots of this structure are: `PIDStringMap` a map that maps the patient id used in the input files onto a new ID used for analysis. `Ctr`: the total number of patients parsed. This counter can be used to generate analysis IDs for patients. `PIDMap`: maps the patient analysis ID onto `Trajectory.Patient` objects. There is also two optional counters for counting the number of males and females.

– The `nofAgegroups` argument are the number of age groups to use when defining cohorts. Can be a CLI parameter.

– The `nofDiagnosisCodes`: the number of different diagnosis codes used in the input.

- `DPatients`: has for each diagnosis the list of patients that are diagnosed with that diagnosis. This can be obtained from the patient lists of the cohorts collected by `trajectory.InitializeCohorts` (see `Cohort.DPatients`).
- `Pairs` and `Trajectories` do not need to be initialized, as they are filled in at later steps.

2. Initialize the experiment relative risk ratios

The relative risk ratios (RR) are initialized by calling the function `trajectory.InitializeExperimentRelativeRiskRatios`. The signature of this function is shown in Listing 9:

```
func InitializeExperimentRelativeRiskRatios(
        exp *Experiment, minTime, maxTime float64, iter int)
```

Listing 9. Signature of the function for initializing relative risk ratios

The parameters are:

- the `trajectory.Experiment` object `exp` created in step 1
- the `minTime` and `maxTime` parameters respectively for the minimum and maximum allowed time between diagnoses to be considered for RR calculation. This is a parameter passed via the CLI.
- the `iter` parameter that determines the number of sampling iterations for calculating the RR. This is a parameter passed via the CLI.

3. Build the experiment's trajectories

The trajectories are built by calling the function `trajectory.BuildTrajectories`. The signature of this function is shown in Listing 10:

```
func BuildTrajectories(exp *Experiment, minPatients,
        maxLength, minLength int, minTime, maxTime, minRR float64,
        filters []TrajectoryFilter) []*Trajectory
```

Listing 10. Signature of the function for building trajectories

The parameters of this function are:

- the `trajectory.Experiment` object `exp` created in step 1
- the `minP` parameter for the minimum number of patients for a trajectory to be considered. This is a parameter passed via the CLI.
- the `minL` and `maxL` parameters respectively for the minimum and maximum length for a trajectory to be considered. These parameters are passed via the CLI.
- the `minT` and `maxT` parameters respectively stand for the minimum and maximum time between trajectory transitions. These parameters are passed via the CLI.
- the `minRR` for the minimum relative risk score (RR) of a diagnosis pair to be considered for building trajectories.

4. Output the found trajectories to disk

The trajectories can be outputted to disk by calling the function `trajectory.PrintTrajectoriesToFile`. This function takes as input the experiment object created in step 1 and an output path.

5. Cluster the trajectories and output the clusters to disk

The trajectories can be clustered by calling the function ‘cluster.ClusterTrajectoriesDirectly‘. The signature of this function is: `func ClusterTrajectoriesDirectly(exp *trajectory.Experiment, granularities []int, path, pathToMcl string)`

The parameters of this function are:

- the `trajectory.Experiment` object `exp` created in step 1.
- the `granularities` parameter: a list of granularities for the clustering step. This is a parameter passed via the CLI.
- the `pathToMCL` parameter: a path to the clustering tool. This parameter is passed via the CLI.
