# Supplementary material for: A software package for efficient patient trajectory analysis applied to analyzing bladder cancer development
Source: PLOS Digit Health. 2023 Nov 22;2(11):e0000384. doi: 10.1371/journal.pdig.0000384 (PMC10664923; doi:10.1371/journal.pdig.0000384)
Supplement: S2 Appendix — This appendix lists all clusters found for the full bladder cancer cohort while generating all found trajectories. (PDF) [file pdig.0000384.s002.pdf]

S2 Appendix: Clusters for full bladder cancer cohort

Charlotte Herzeel[1], Ellie D'Hondt[1], Valerie Vandeweerd[2], Wouter Botermans[2], Murat Akand[3], Frank Van der Aa[3], Roel Wuyts[1], Wilfried Verachtert[1],

1 ExaScience Lab, imec, Leuven, Belgium
2 Janssen Research & Development, a division of Janssen Pharmaceutica NV, Beerse, Belgium
3 Department of Urology, University Hospitals Leuven, Leuven, Belgium

* Charlotte.Herzeel@imec.be

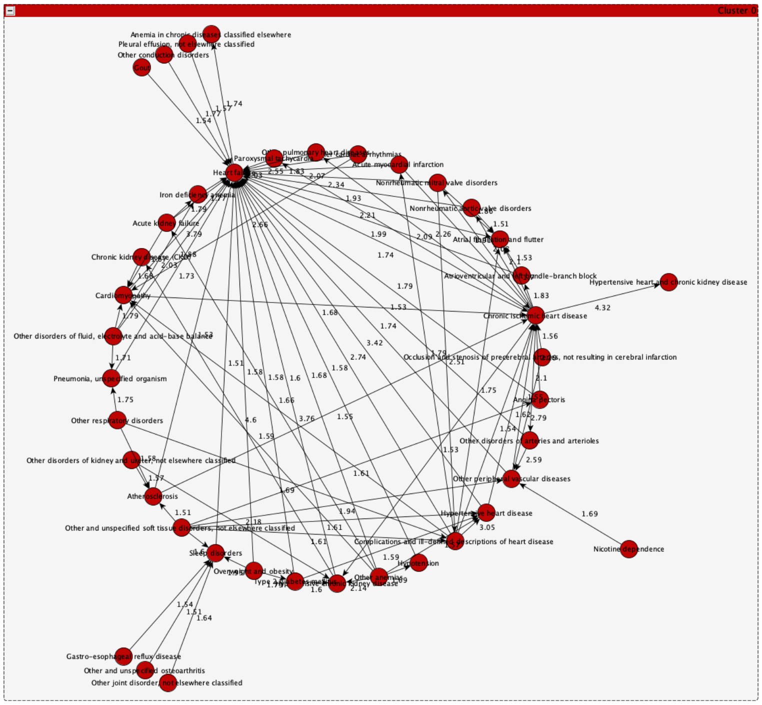

Fig A. Cluster 0: Heart disease

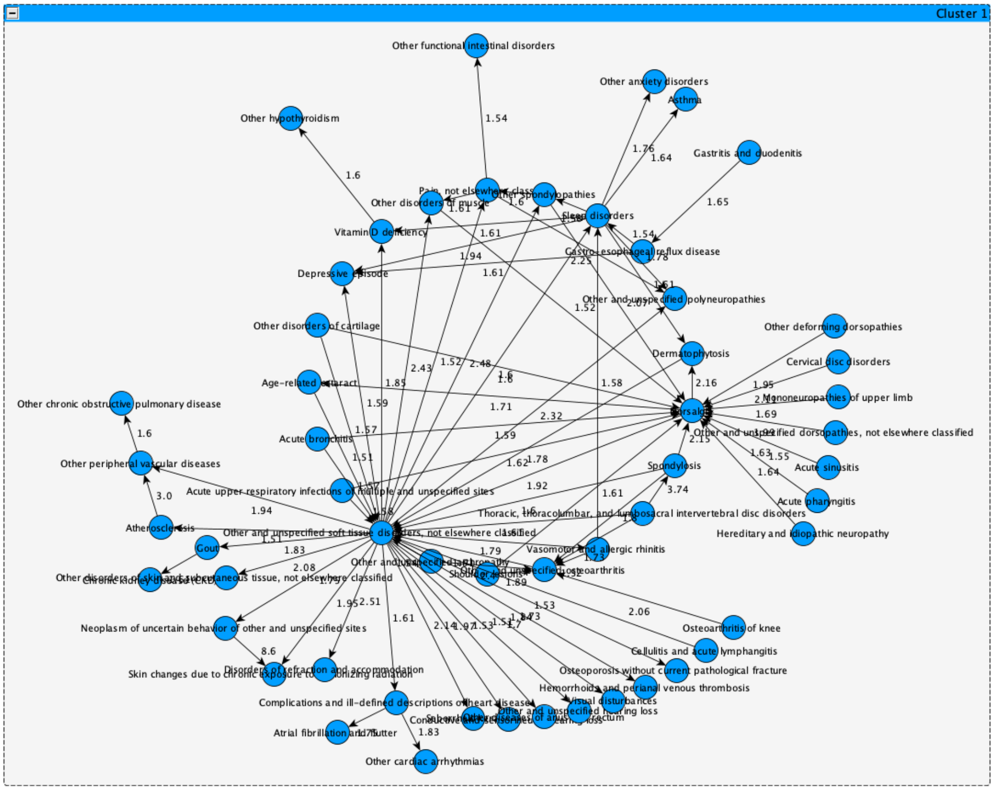

Fig B. Cluster 1: Systemic disease

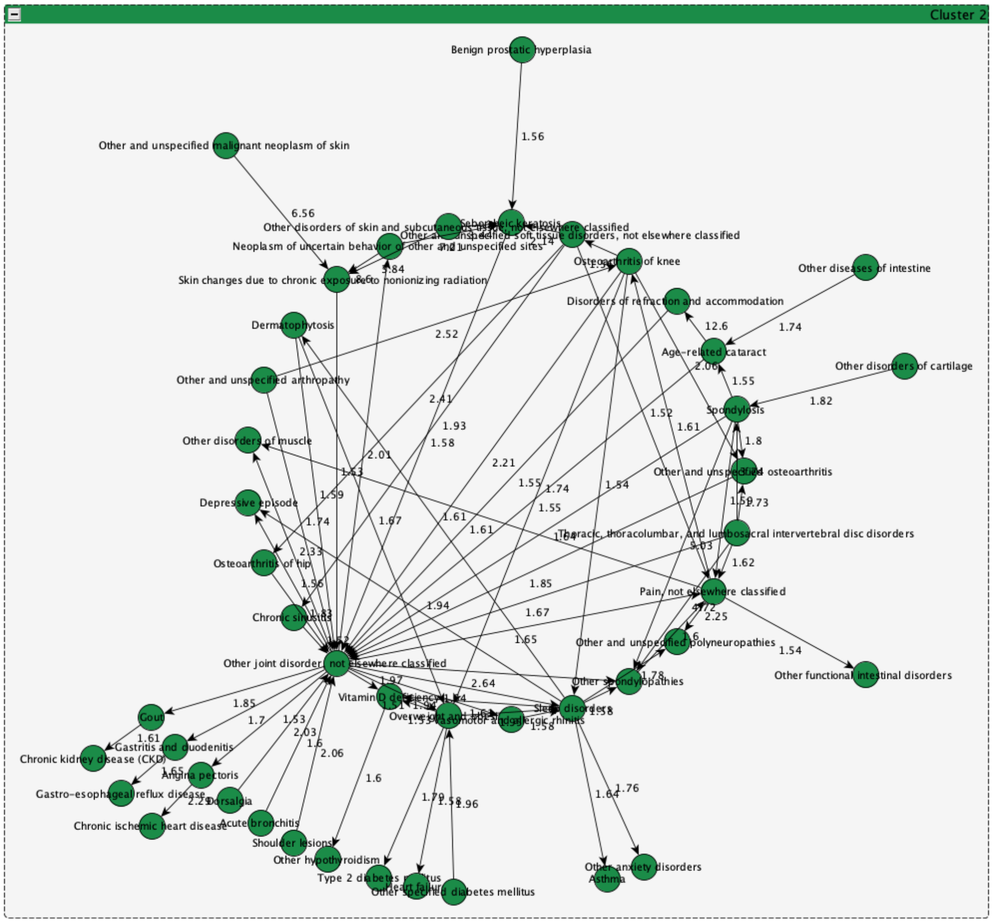

Fig C. Cluster 2: Unknown

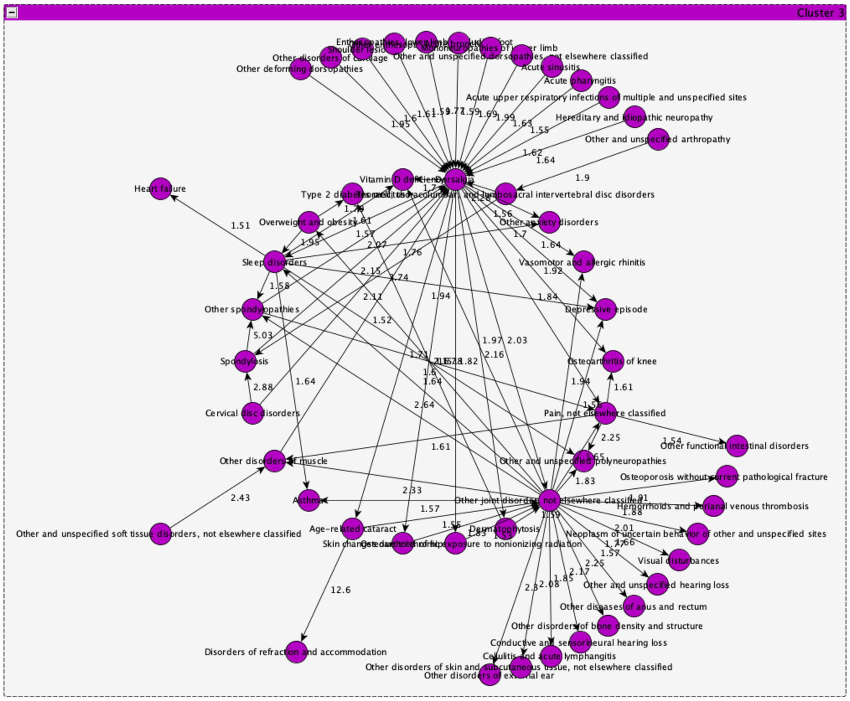

Fig D. Cluster 3: Musculoskeletal disorders

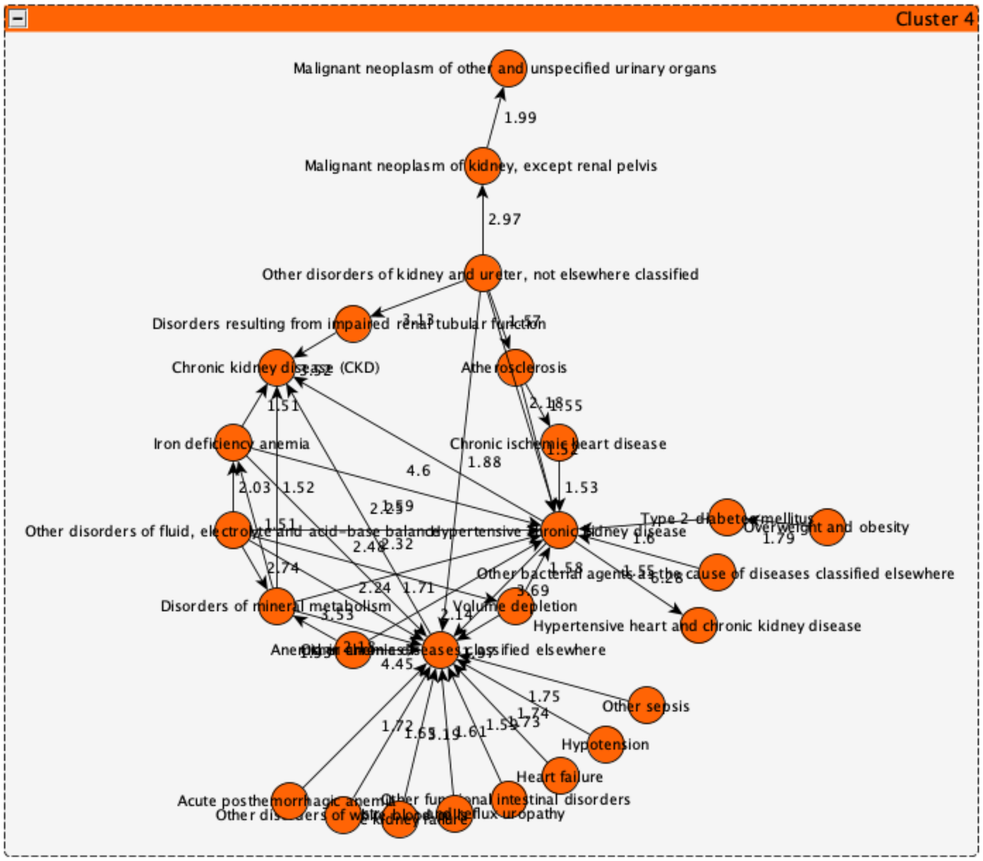

Fig E. Cluster 4: Cardiorenal disease

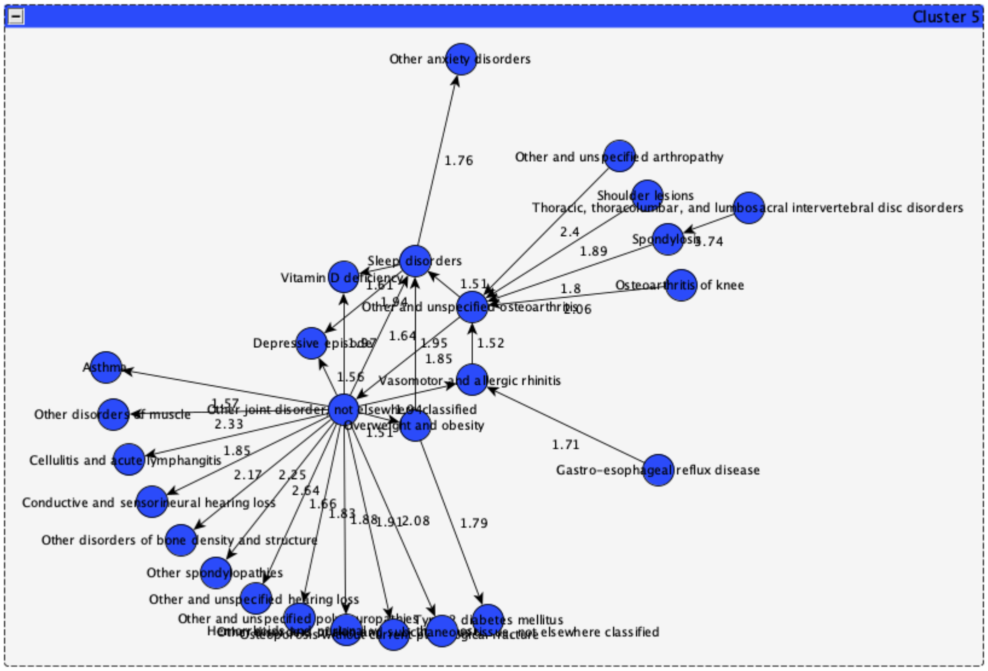

Fig F. Cluster 5: Sleep/anxiety disorder

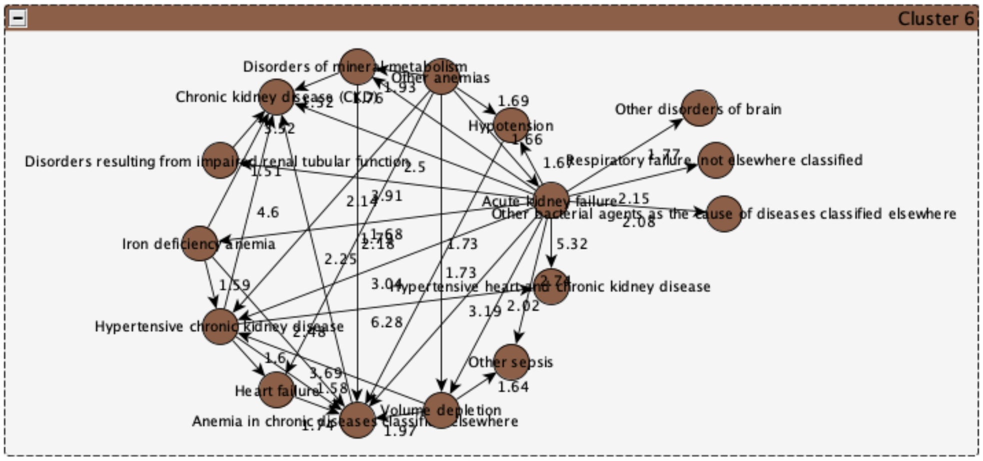

Fig G. Cluster 6: Kidney disease

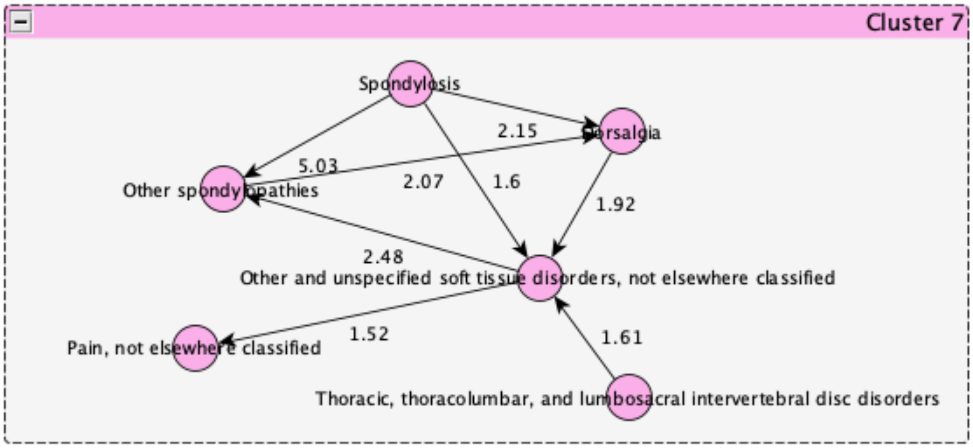

Fig H. Cluster 7: Dorsopathy

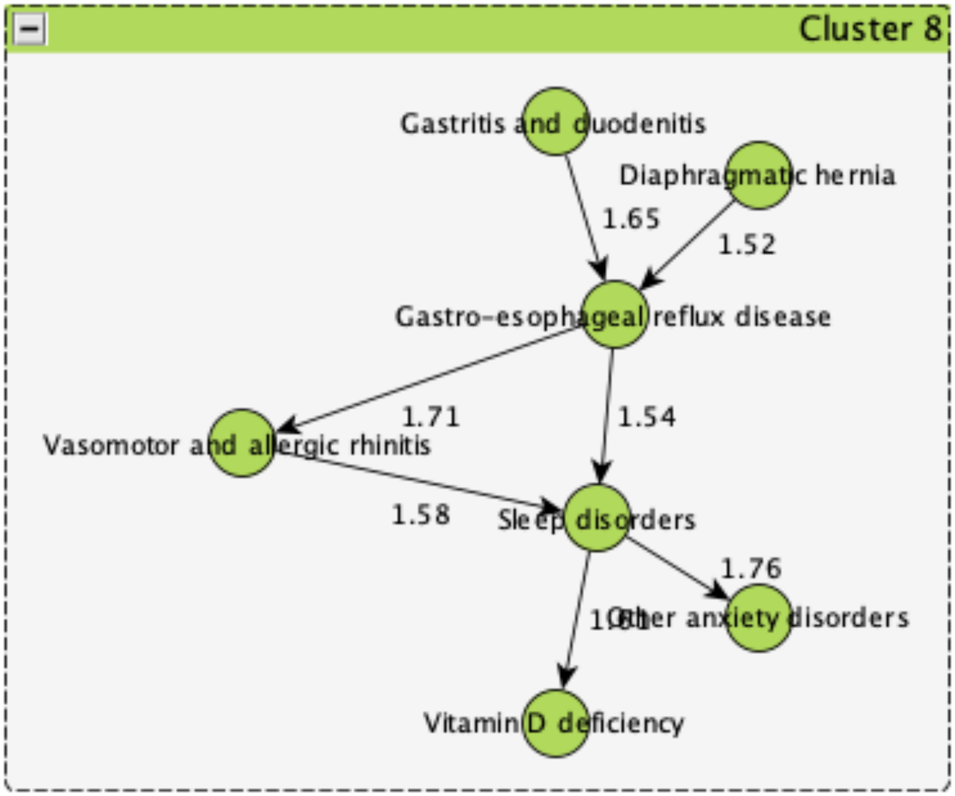

Fig I. Cluster 8: Sleep/Anxiety disorder

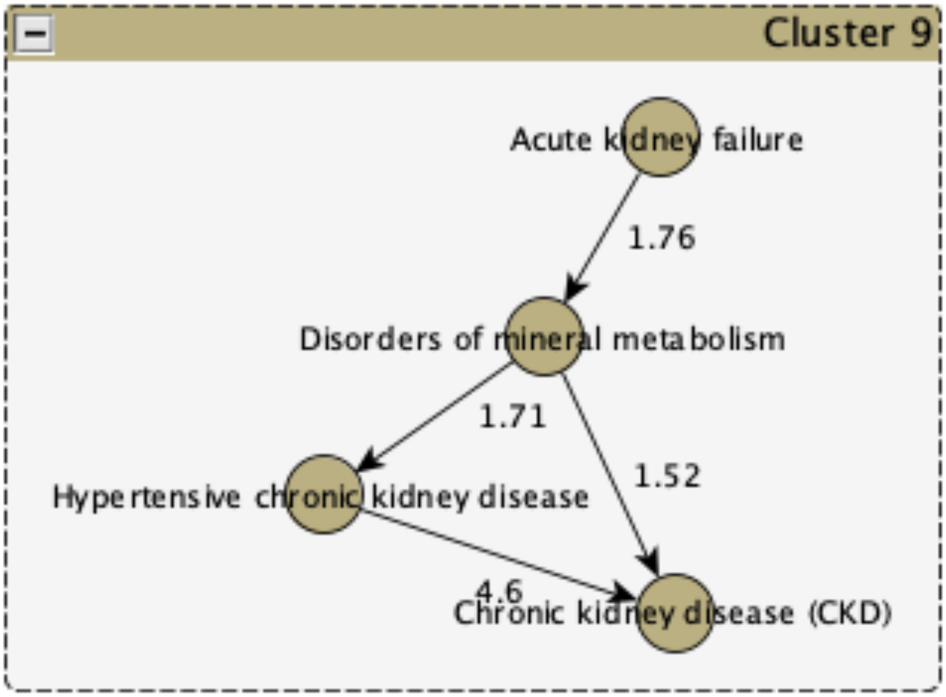

Fig J. Cluster 9: Kidney disease

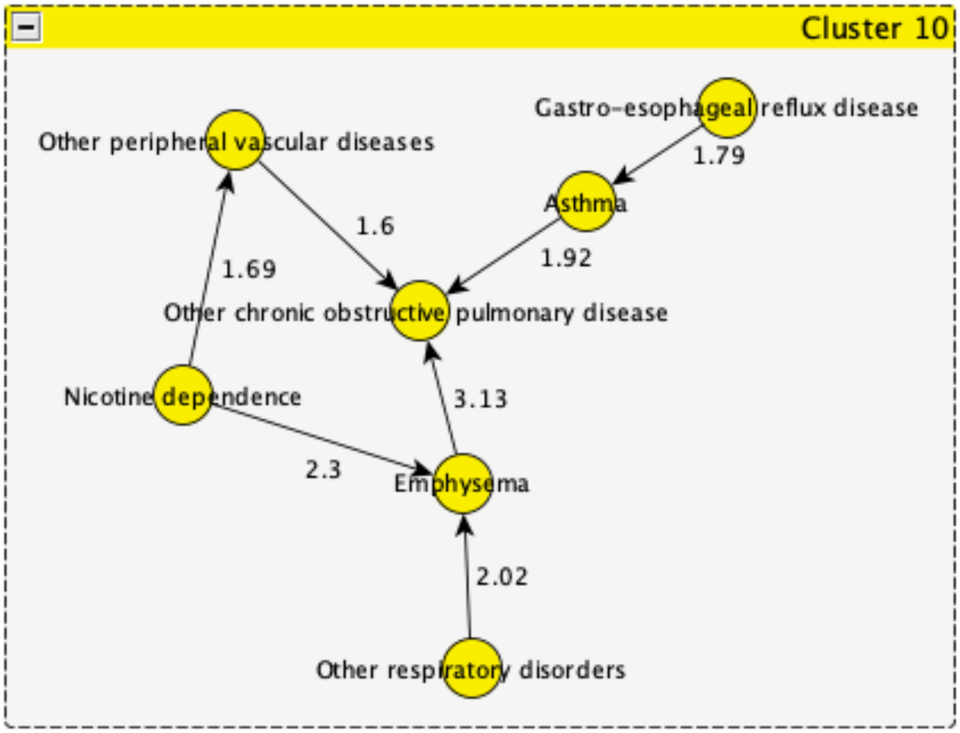

Fig K. Cluster 10: Lung disease (chronic issues)

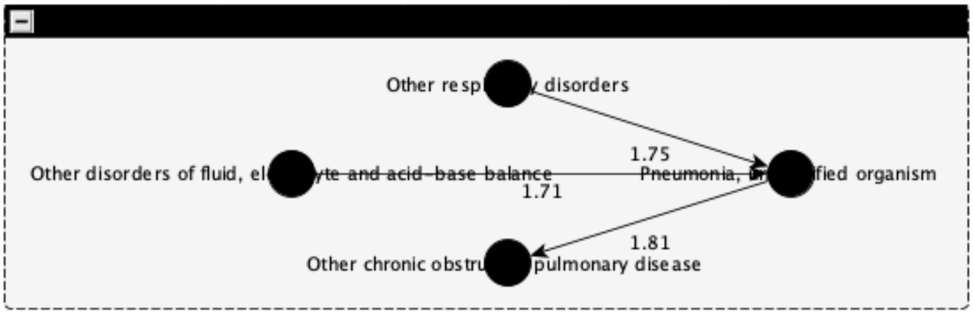

Fig L. Cluster 11: Lung disease (infection)

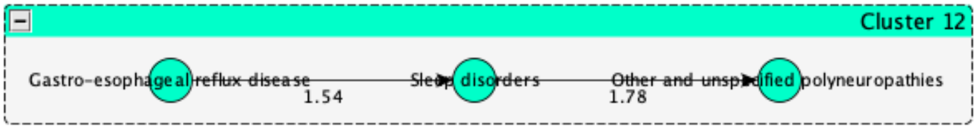

Fig M. Cluster 12: Unknown

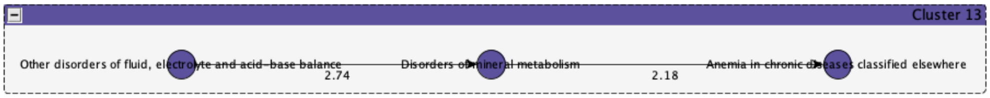

Fig N. Cluster 13: Blood chemistry deviations

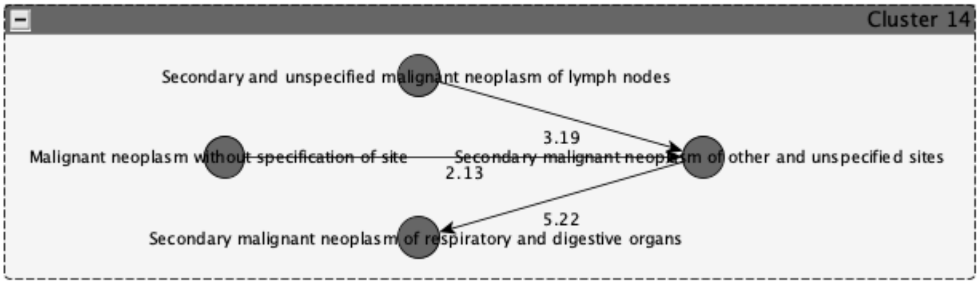

Fig O. Cluster 14: Cancer

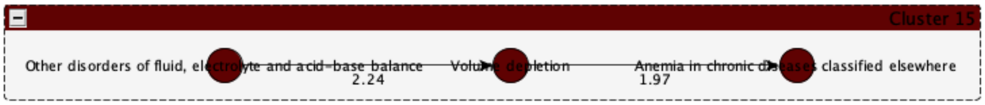

Fig P. Cluster 15: Blood chemistry deviations

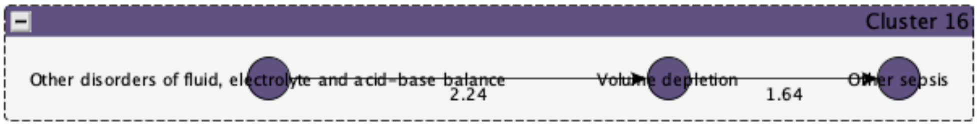

Fig Q. Cluster 16: Blood chemistry deviations

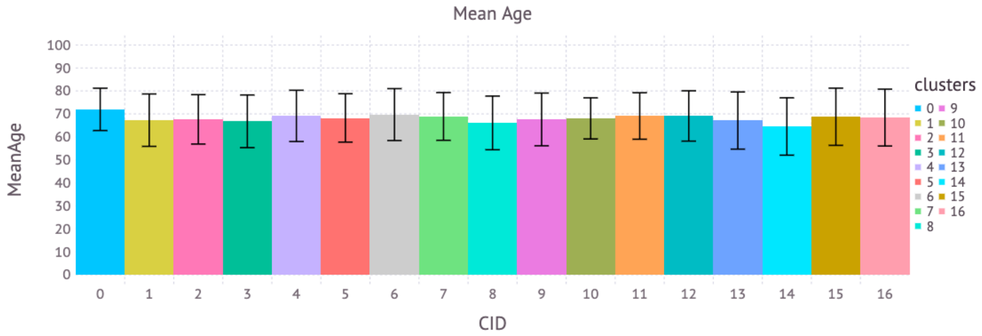

Fig R. Mean age per cluster.

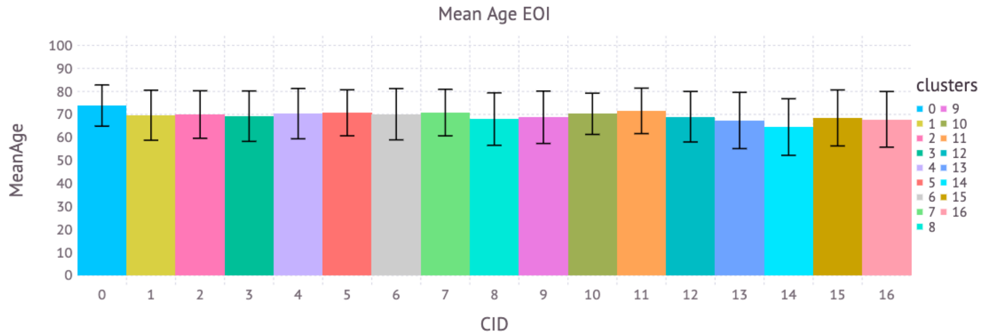

Fig S. Mean age at the time of event of interest (bladder cancer) per cluster.

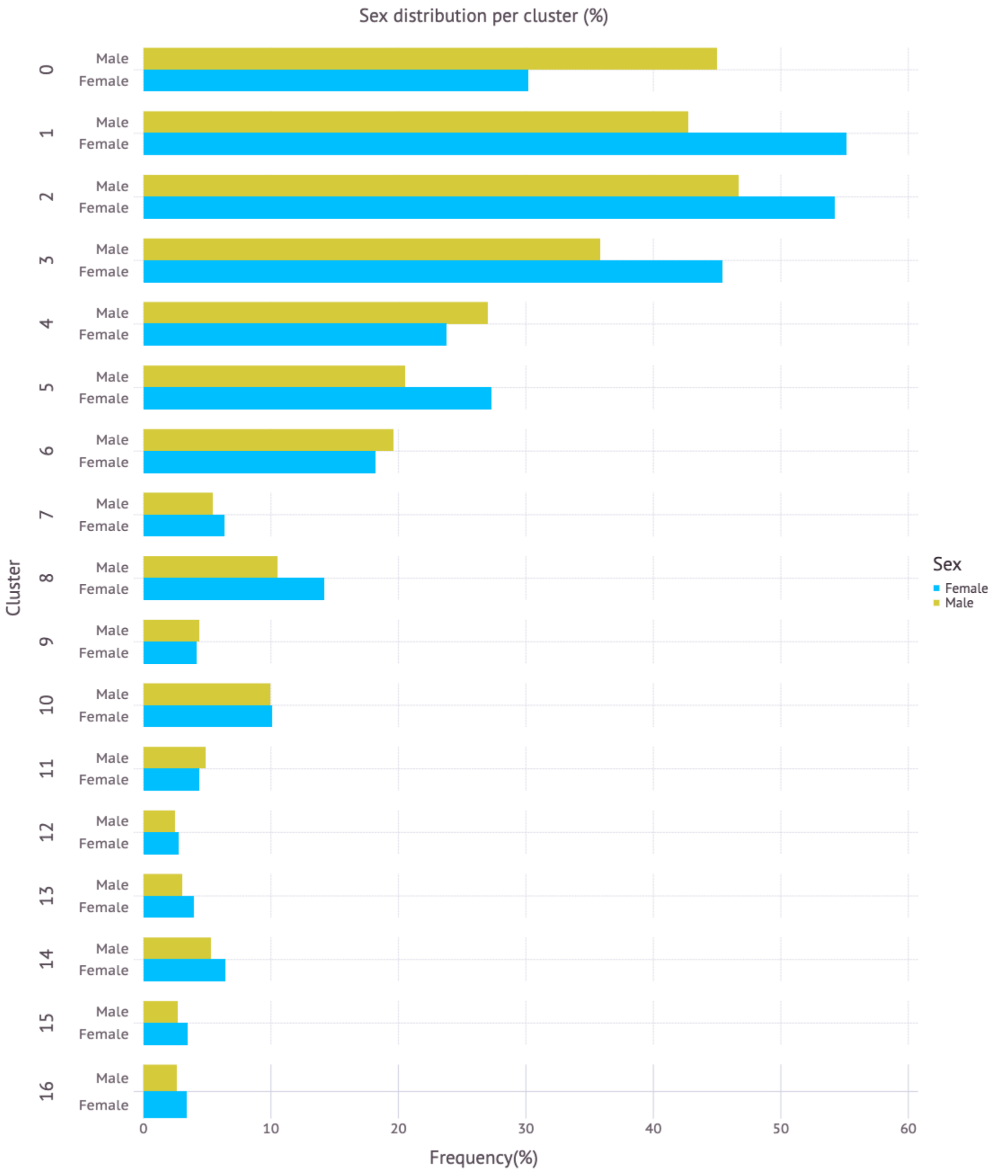

Fig T. Sex distribution (proportion of males/females) per cluster.
