# Supplementary material for: A software package for efficient patient trajectory analysis applied to analyzing bladder cancer development
Source: PLOS Digit Health. 2023 Nov 22;2(11):e0000384. doi: 10.1371/journal.pdig.0000384 (PMC10664923; doi:10.1371/journal.pdig.0000384)
Supplement: S3 Appendix — This appendix lists all bladder cancer-specific clusters found for the full bladder cancer cohort, using the TriNetX ICD diagnosis codes, as well as bladder cancer-specific treatments. The trajectories shown are filtered to only show trajectories where at least one medical event is related to cancer. (PDF) [file pdig.0000384.s003.pdf]

S3 Appendix: Bladder cancer-specific clusters

Charlotte Herzeel[1], Ellie D'Hondt[1], Valerie Vandeweerd[2], Wouter Botermans[2], Murat Akand[3], Frank Van der Aa[3], Roel Wuyts[1], Wilfried Verachtert[1],

1 ExaScience Lab, imec, Leuven, Belgium
2 Janssen Research & Development, a division of Janssen Pharmaceutica NV, Beerse, Belgium
3 Department of Urology, University Hospitals Leuven, Leuven, Belgium

* Charlotte.Herzeel@imec.be

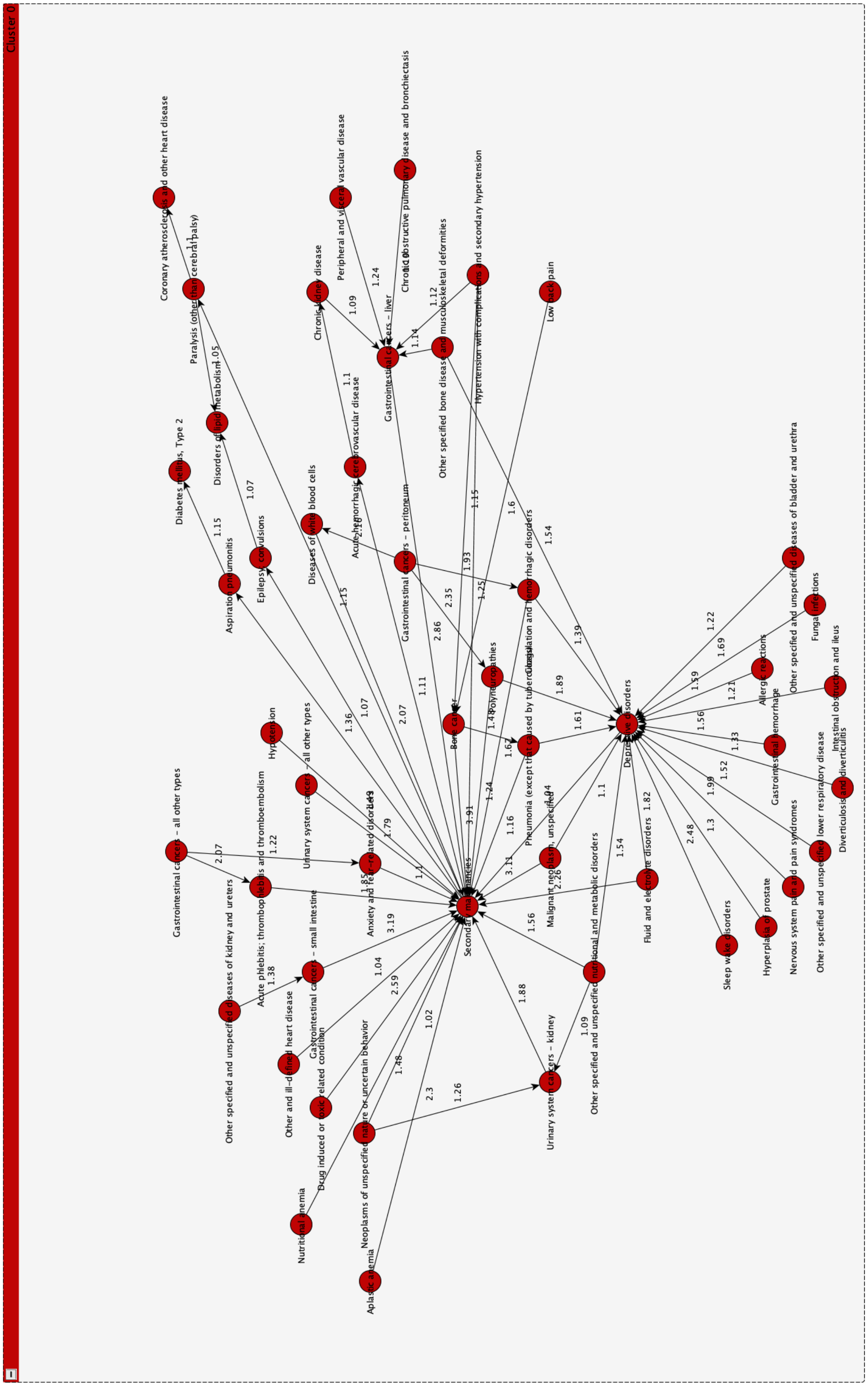

Fig A. Cluster 0: Secondary malignancy

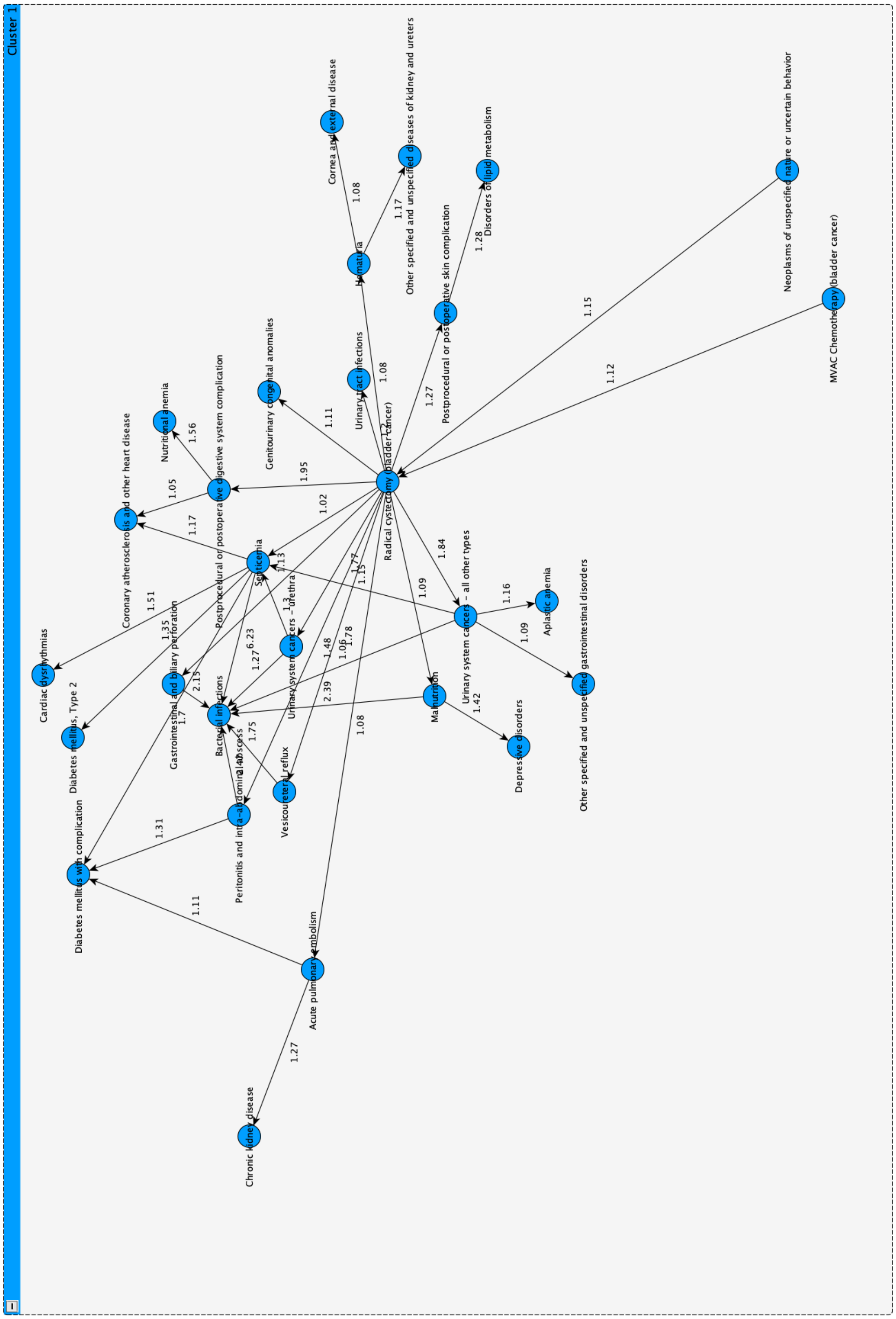

Fig B. Cluster 1: Radical cystectomy

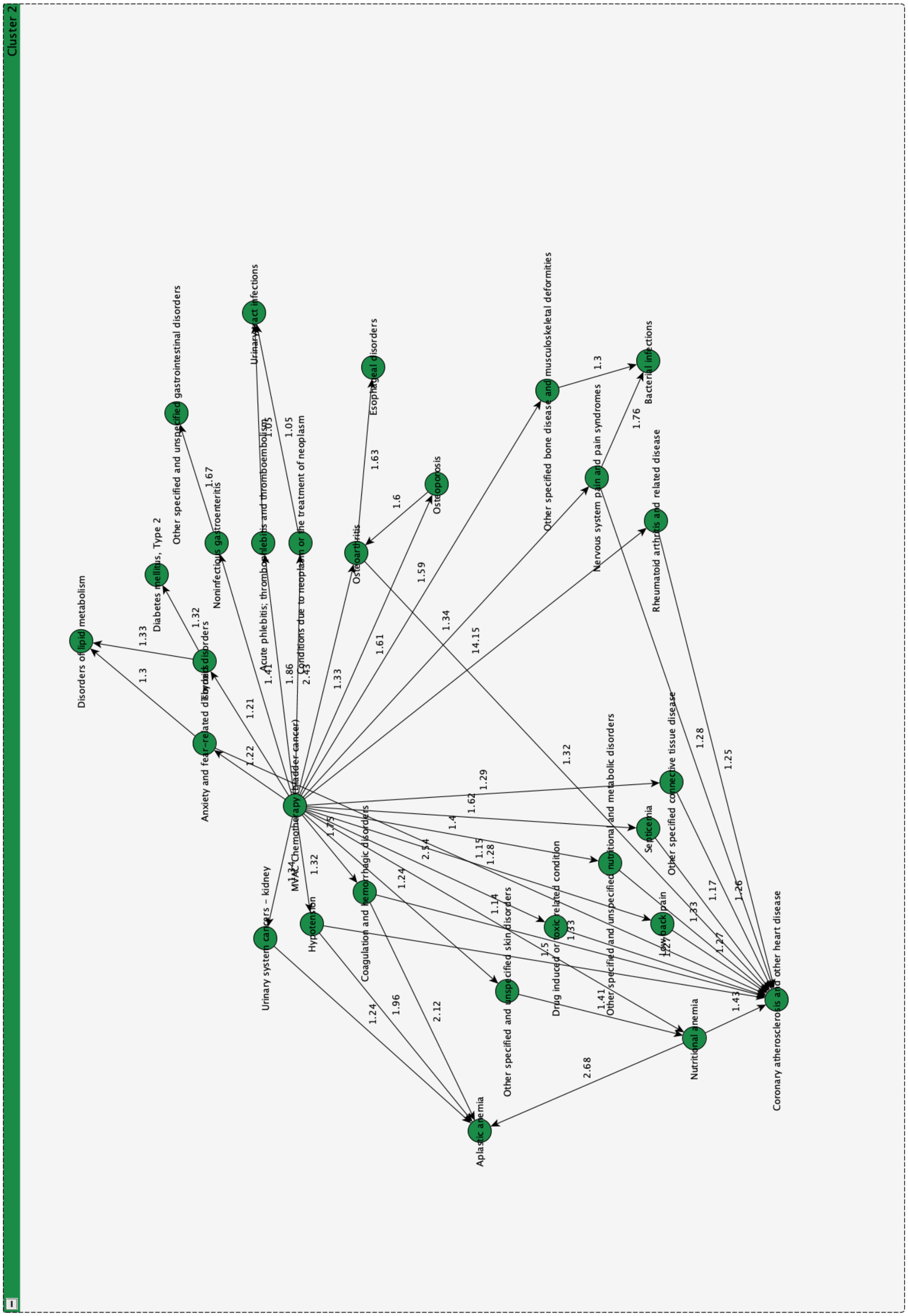

Fig C. Cluster 2: MVAC chemotherapy

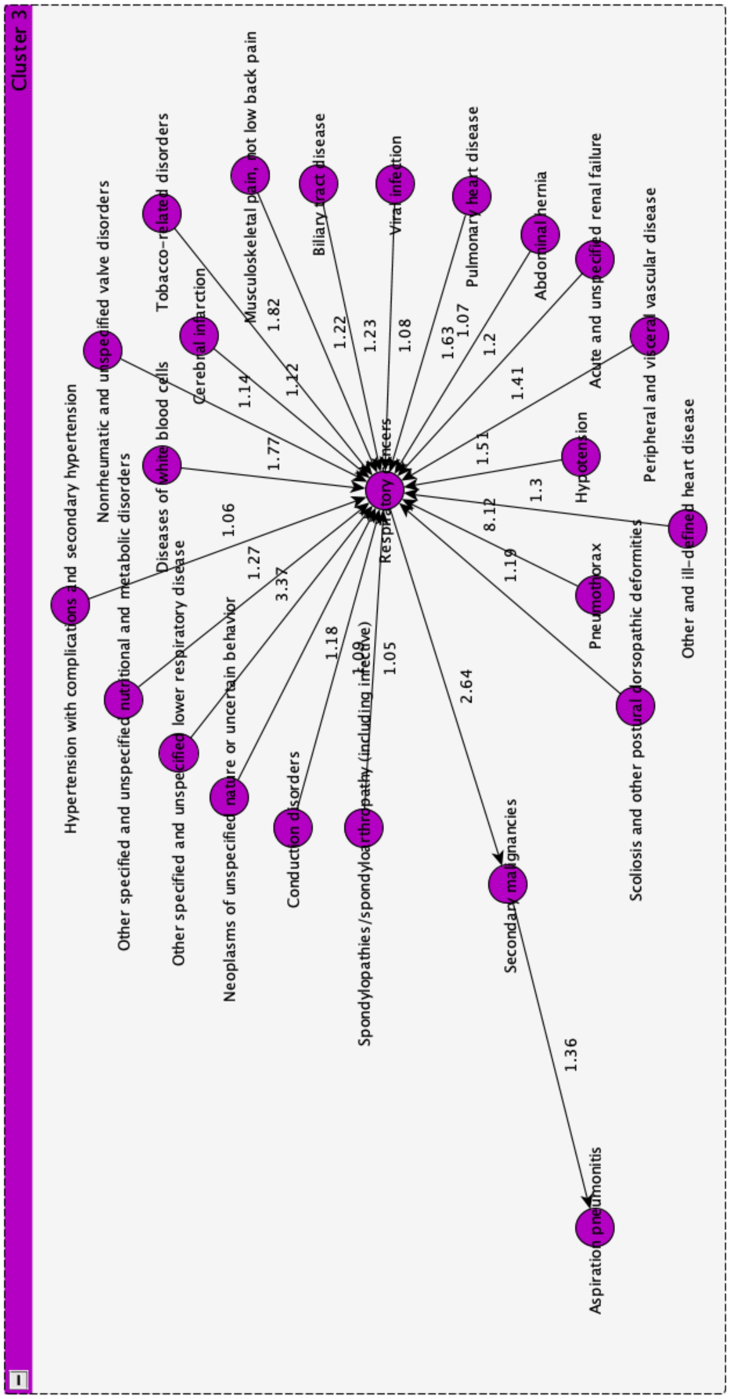

Fig D. Cluster 3: Secondary malignancy

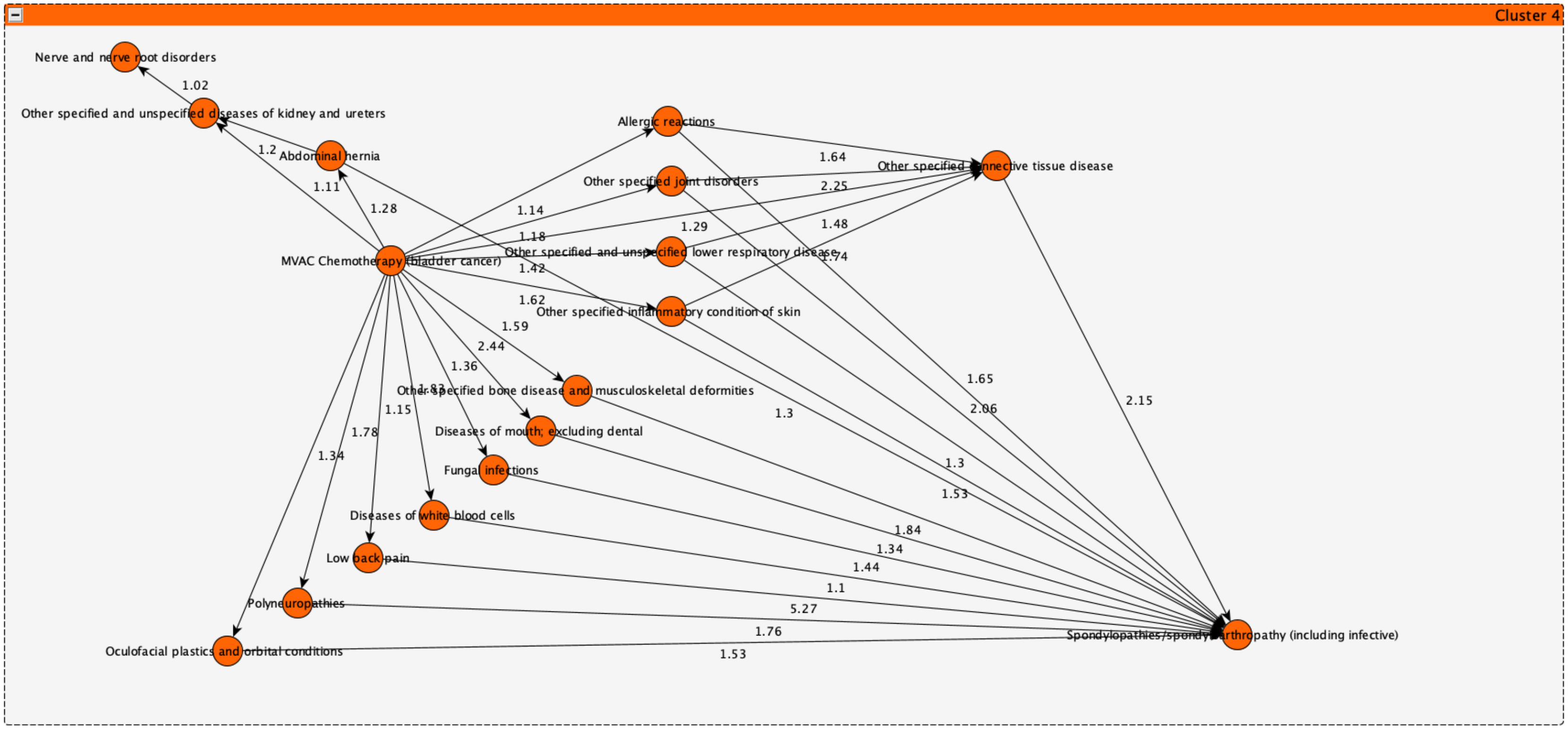

Fig E. Cluster 4: MVAC chemotherapy

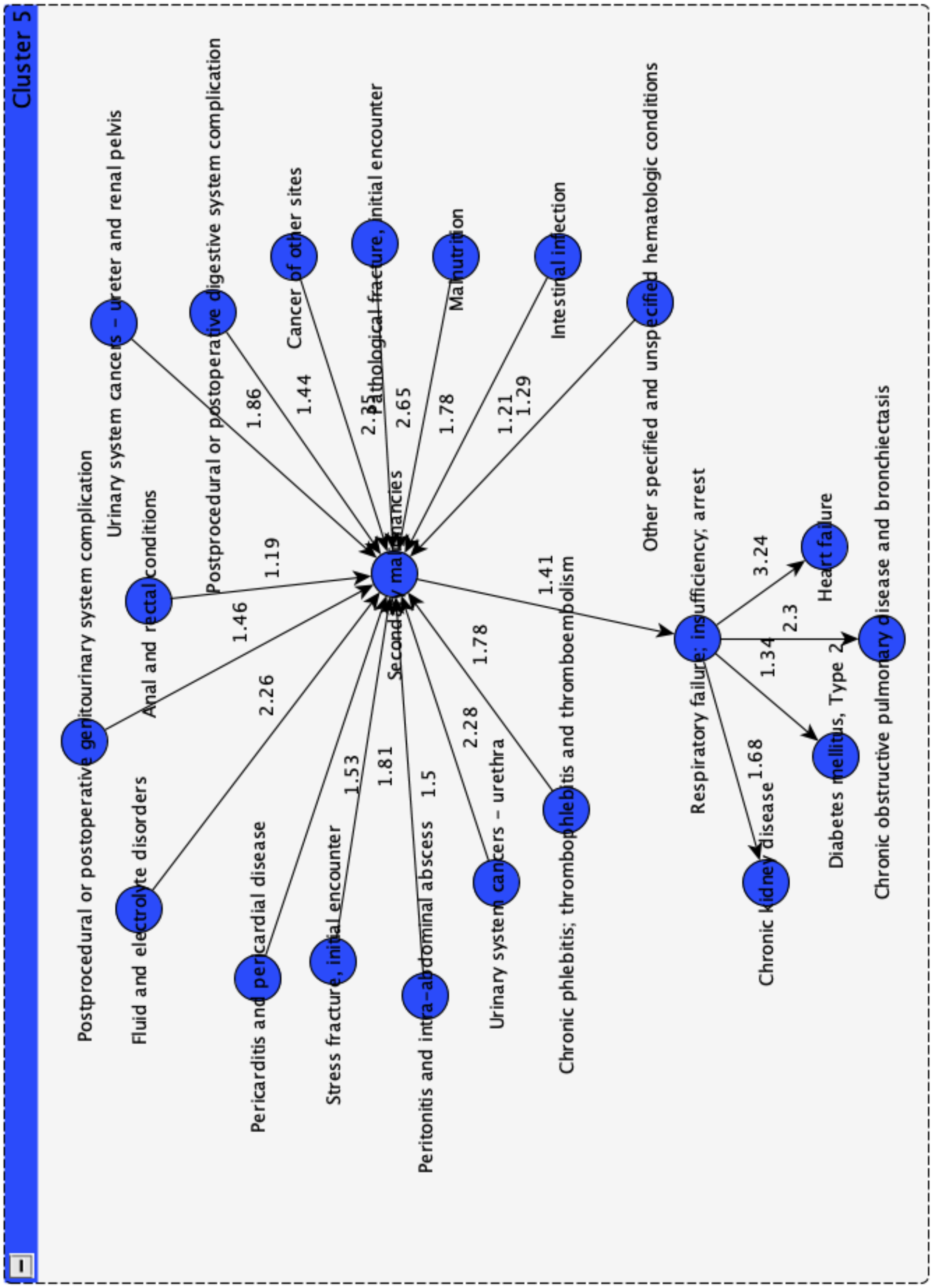

Fig F. Cluster 5: Secondary malignancy

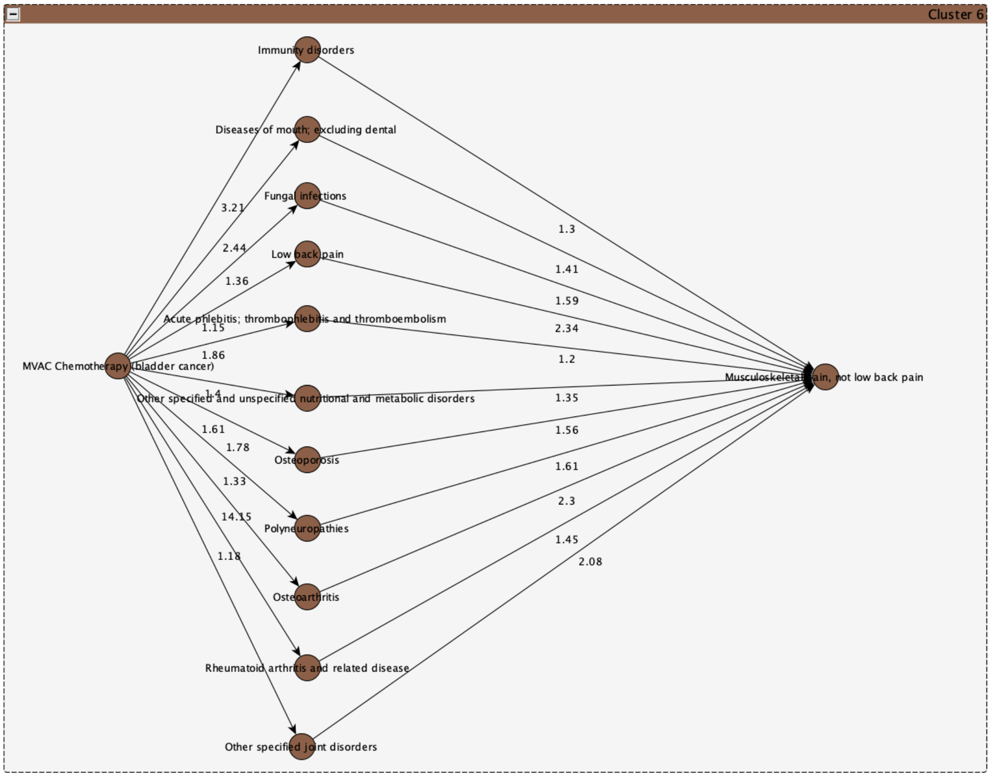

Fig G. Cluster 6: MVAC chemotherapy

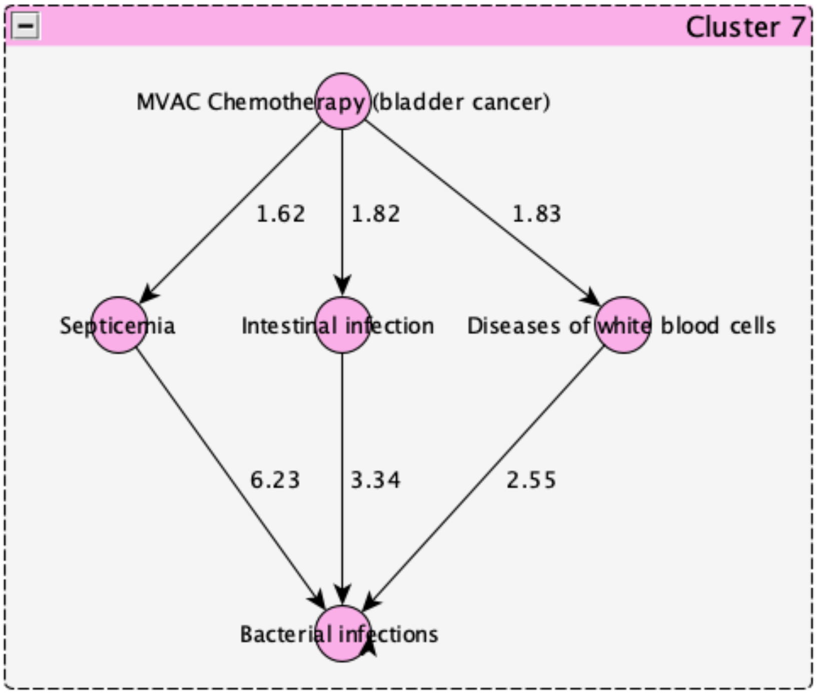

Fig H. Cluster 7: MVAC chemotherapy

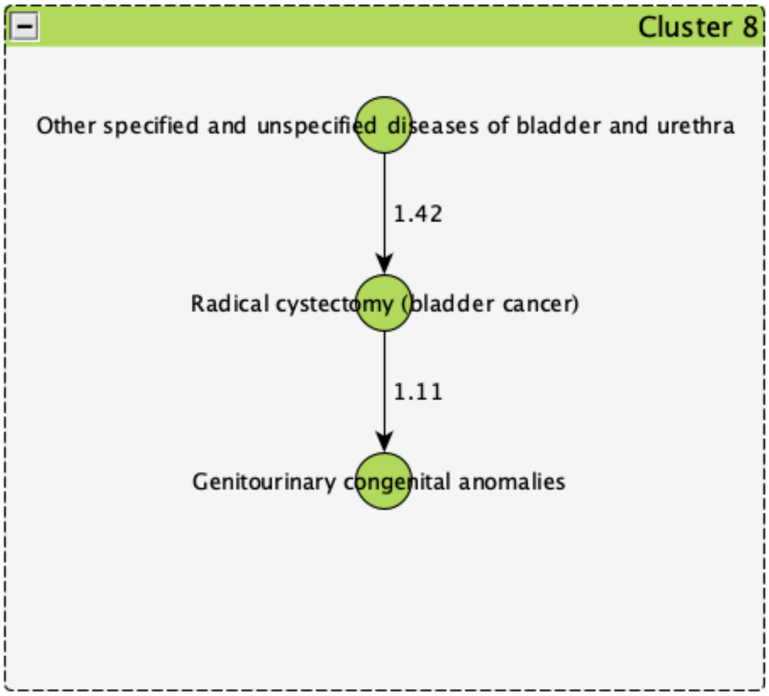

Fig I. Cluster 8: Radical cystectomy

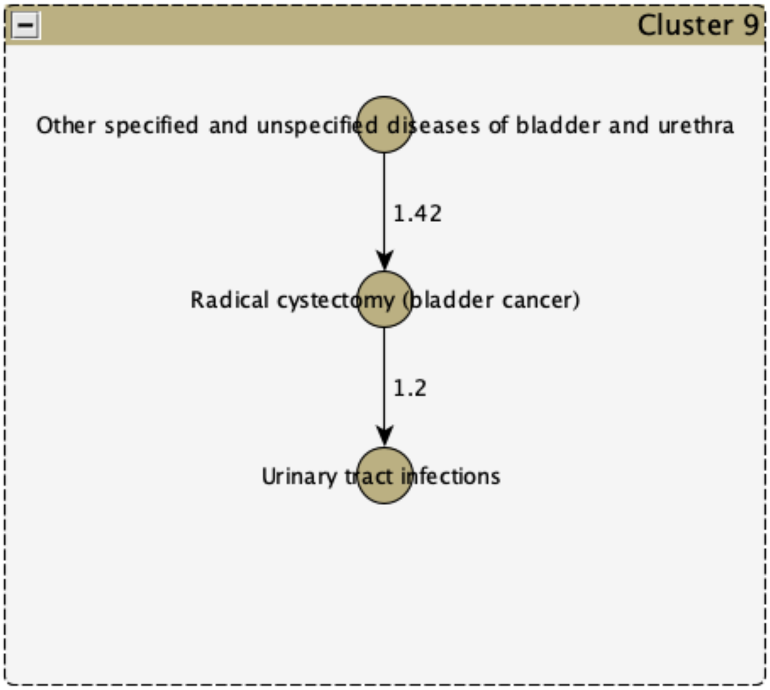

Fig J. Cluster 9: Radical cystectomy

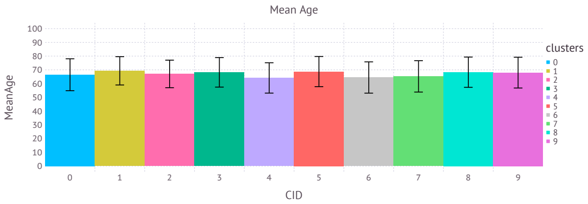

Fig K. Mean age per cluster.

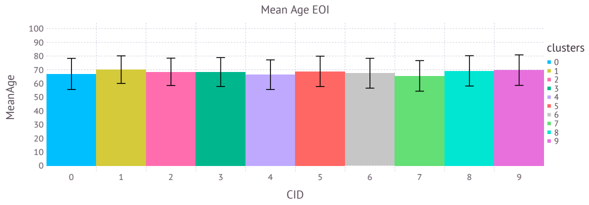

Fig L. Mean age at the time of event of interest (bladder cancer) per cluster.

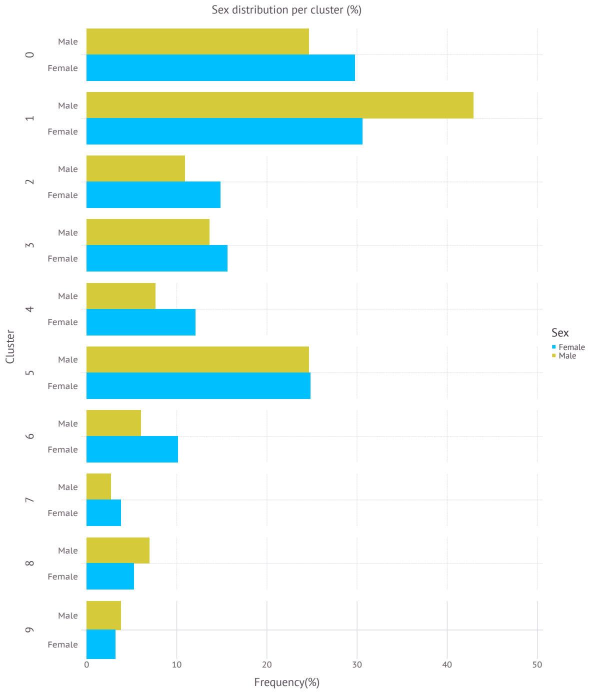

Fig M. Sex distribution (proportion of males/females) per cluster.
